# Serological and transcriptomic response of African fruit bats to immune challenge with recombinant vaccines expressing Ebola or Nipah virus antigens

**DOI:** 10.1098/rsos.252189

**Published:** 2026-10-07

**Authors:** Elinor Jax, Romain Garnier, Georgia Tsagkogeorga, Hubert Buczkowski, Louise Gibson, Pius Benia, Richard Suu-Ire, Vera M. Warmuth, Sylvester Languon, Osbourne Quaye, Lianying Yan, Christopher C. Broder, Stephen J. Rossiter, Edward Wright, Sarah C. Gilbert, Teresa Lambe, James L. N. Wood, Andrew A. Cunningham, Olivier Restif

**Affiliations:** ^1^ Department of Veterinary Medicine, University of Cambridge, Cambridge, UK; ^2^ School of Biological and Behavioural Sciences, Queen Mary University of London, London, UK; ^3^ Viral Pseudotype Unit, University of Sussex, Brighton, UK; ^4^ Institute of Zoology of the Zoological Society of London, London, UK; ^5^ School of Veterinary Medicine, College of Basic and Applied Sciences, University of Ghana, Accra, Ghana; ^6^ Department of Biochemistry, Cell and Molecular Biology, West African Centre for Cell Biology of Infectious Pathogens, University of Ghana, Accra, Ghana; ^7^ Biozentrum Martinsried, Ludwig-Maximilians-Universität München, Planegg-Martinsried, Germany; ^8^ Department of Microbiology and Immunology, Uniformed Services University, Bethesda, MD, USA; ^9^ Henry M Jackson Foundation for the Advancement of Military Medicine Inc, Rockville, MD, USA; ^10^ University of Oxford Pandemic Sciences Institute, Oxford, UK; ^11^ Chinese Academy of Medical Sciences (CAM) Oxford Institute, University of Oxford, Oxford, UK; ^12^ Oxford Vaccine Group, University of Oxford, Oxford, UK

**Keywords:** *Eidolon helvum*, *Epomophorus gambianus*, henipavirus, Paramyxoviridae, Filoviridae, gene expression, antibody production

## Abstract

Bats are hosts to diverse zoonotic viruses which can cause high case fatality rates in humans. Since detecting viruses in bats is difficult, antibodies are often used for surveillance, though their significance remains unclear in the absence of *in vivo* immunological studies. To address this knowledge gap, we measured antibody production and gene expression in bats following injection with vaccines encoding either a Nipah virus G glycoprotein or an Ebola virus GP glycoprotein. We selected two African bat species with overlapping ranges, *Eidolon helvum* (confirmed reservoir of henipaviruses) and *Epomophorus gambianus* (putative reservoir of filoviruses). Using a Luminex-based multiplex microsphere immunoassay and a pseudotype virus neutralization assay, we observed an increase in antibodies against the Nipah glycoprotein in most bats that received the Nipah vaccine. In contrast, we could only detect anti-Ebola glycoprotein antibodies in one bat species, *Ep. gambianus*, in response to the Ebola vaccine. Transcriptomic analysis in *Ei. helvum* revealed that genes involved in ISGylation and T-cell differentiation pathways were differentially expressed in response to the Ebola, but not the Nipah, vaccine. These results demonstrate species-specific differences in responses to antigen exposure, offering insights into bat adaptive immune systems and providing calibration for serological data from wild bats.

## 1. Introduction

The role of bats as natural reservoirs for emerging zoonotic viruses has become a major concern in the last 20 years [1,2]. This has motivated unprecedented research effort into bat immunology, with approaches including experimental infections in high-containment facilities, the development of bat cell lines for *in vitro* immunoassays and bioinformatic analysis of bat genome and transcriptome sequences [3]. Recent investigations have identified unique adaptations of the innate immune system in bats which may explain how they can host viruses with high mortality in other species without presenting clinical disease [4]. In contrast, the adaptive immune system of bats remains poorly understood [3].

Virus-specific antibody measurement is routinely used as a marker of viral exposure in wild-caught bats and plays a crucial role in the surveillance of wildlife pathogens of zoonotic risk [5]. However, interpreting these assays and inferring seroprevalence in bat populations is hindered by a lack of understanding of the kinetics and function of antibodies during or after viral infection in bats [3,6]. A review of experimental infection and vaccination in bats noted that antibody responses were variable, with some animals remaining immune to viral challenge even in the absence of detectable neutralizing antibodies [7]. In addition, results from experimental infections appear to vary between bat species and viral infections [8]. More detailed information on kinetics, magnitude and functionality of the humoral immune response in different bat species would facilitate the interpretation of serological data from the wild [9].

Henipaviruses (family Paramyxoviridae) [10–14] and Ebola viruses (family Filoviridae) [15–21] have caused repeated deadly outbreaks in people and domestic animals on three continents [22–25], and both groups have been linked to fruit bat hosts from the Pteropodidae family. This family consists of nearly 200 species that are widely distributed in tropical regions in Africa, Asia, Australia and many Pacific and Indian Ocean islands [26]. Serological data from wild bats have revealed differences in antibody levels against henipaviruses and filoviruses in fruit bat species with overlapping distributions [19,27,28], yet little is known about the mechanisms behind host-specificity (but see [29]).

Experimental infections with henipaviruses in a few bat species have confirmed the production of serum antibodies and the excretion of virus in urine without developing apparent clinical disease [14,30–32]. Experimental infections with Ebola viruses in the Egyptian fruit bat (*Rousettus aegyptiacus*) likewise yielded antibodies without apparent clinical disease, but no viraemia was recorded aside from low levels of viral RNA detection [33,34]. The authors concluded that this bat species is an unlikely source of Ebola virus spillover in nature. While focusing on virus and antibody detection, none of these studies provided further characterization of the immune response of live bats following viral exposure. Extending these experimental studies to other pairs of bat and virus species is made extremely difficult and costly by biocontainment imperatives, creating a need for alternative approaches.

As a new step towards unravelling the kinetics of the adaptive antiviral immune response of bats *in vivo*, we designed an experiment with two African fruit bat species, using recombinant vaccines against Ebola and Nipah viruses as a tool to alleviate the biosafety constraints inherent to the study of filoviruses and henipaviruses. The two bat species, *Eidolon helvum* and *Epomophorus gambianus*, were chosen for their documented association with henipaviruses and filoviruses, respectively. The straw-coloured fruit bat, *Ei. helvum*, spans most of subtropical Africa, forming large, migratory and panmictic colonies [35–37], with estimates of seroprevalence against henipaviruses around 40% across its range [36] and repeated detection of viral RNA from multiple paramyxoviruses [36,38]. Antibodies against Ebola virus have also been detected in *Ei. helvum* [17,20,21,39–41], but the interpretation remains speculative in the absence of further validation. The Gambian epauletted fruit bat, *Ep. gambianus*, while less gregarious, is often sympatric with *Ei. helvum* [42]. Detection of paramyxovirus RNA or antibodies is rare in this species [38,43,44]. Antibodies against filoviruses have been detected in wild *Ep. gambianus* on several occasions [18,21,39,41], but active infection has never been confirmed. To date, no filovirus RNA has been found in either bat species, and the sensitivity and specificity of serological assays for antibodies against Ebola virus in bats remain to be assessed. These two species of fruit bat thus provide a unique system to study host–virus specificity: despite overlapping geographic distributions, serological surveys suggest they may be hosts to different subsets of zoonotic viruses and might, therefore, respond differently to virus inoculation.

The objectives of this study were twofold. First, we wanted to compare the dynamics of antibody production in the two bat species in response to vaccine exposure, as a way to improve the interpretation of bat virus serological data from field studies. We hypothesized that recombinant vaccines expressing Nipah or Ebola virus antigens (which had only just become available for research purposes at the time of planning this study [45,46]) would mimic virus inoculation without the need to use live virus, eliciting antibody production comparable to levels observed in the field and revealing differences in virus-specific response between bat species. Our second objective was to identify adaptive immune pathways involved in antiviral response and antibody production in bats, by measuring changes in gene expression for four weeks in a subset of *Ei. helvum* bats from each vaccine group.

## 2. Experimental set-up

Here, we outline the experimental set-up, focusing on the animal work; the full ‘Material and methods’ are presented in §5. After capture and selection of healthy adult males, 19 *Ei. helvum* and 14 *Ep. gambianus* bats were individually tagged with intradermal microchips and were placed in cages (80 × 80 × 80 cm) in single-species groups of up to five bats for the rest of the study (two months). Bats from each species were split into three groups to receive one of three treatments: Ebola vaccine, Nipah vaccine or saline injections. The first group (*n_Eidolon_* = 8, *n_Epomophorus_* = 6) received an intramuscular dose of recombinant chimpanzee adenovirus vaccine (ChAdOx1) encoding the *Orthoebolavirus zairense* glycoprotein (hereafter Ebola virus GP protein), followed four weeks later by an intramuscular dose of modified vaccinia Ankara (MVA) vaccine encoding the same Ebola virus GP protein, following a previously published protocol [47]. The second group (*n_Eidolon_* = 7, *n_Epomophorus_* = 5) received an intramuscular dose of recombinant chimpanzee adenovirus vaccine (ChAdOx1) encoding the *Henipavirus nipahense* G glycoprotein (hereafter Nipah virus G protein), followed four weeks later by an intramuscular dose of MVA vaccine with no Nipah virus antigen (owing to unforeseen last-minute technical difficulties with vaccine preparation; see §5). The third group (*n_Eidolon_* = 4, *n_Epomophorus_* = 3) received two doses of phosphate-buffered saline (PBS) four weeks apart. Bat numbers and timelines are shown in figure 1. We experienced unexpectedly high attrition among *Epomophorus* bats in all three groups (unrelated to the vaccine injection), with only four bats surviving the first four weeks and three to the end (see §5 for further explanations). Blood samples from surviving bats were collected weekly, and organs were harvested following euthanasia at the end of the experiment.

**Figure 1.**
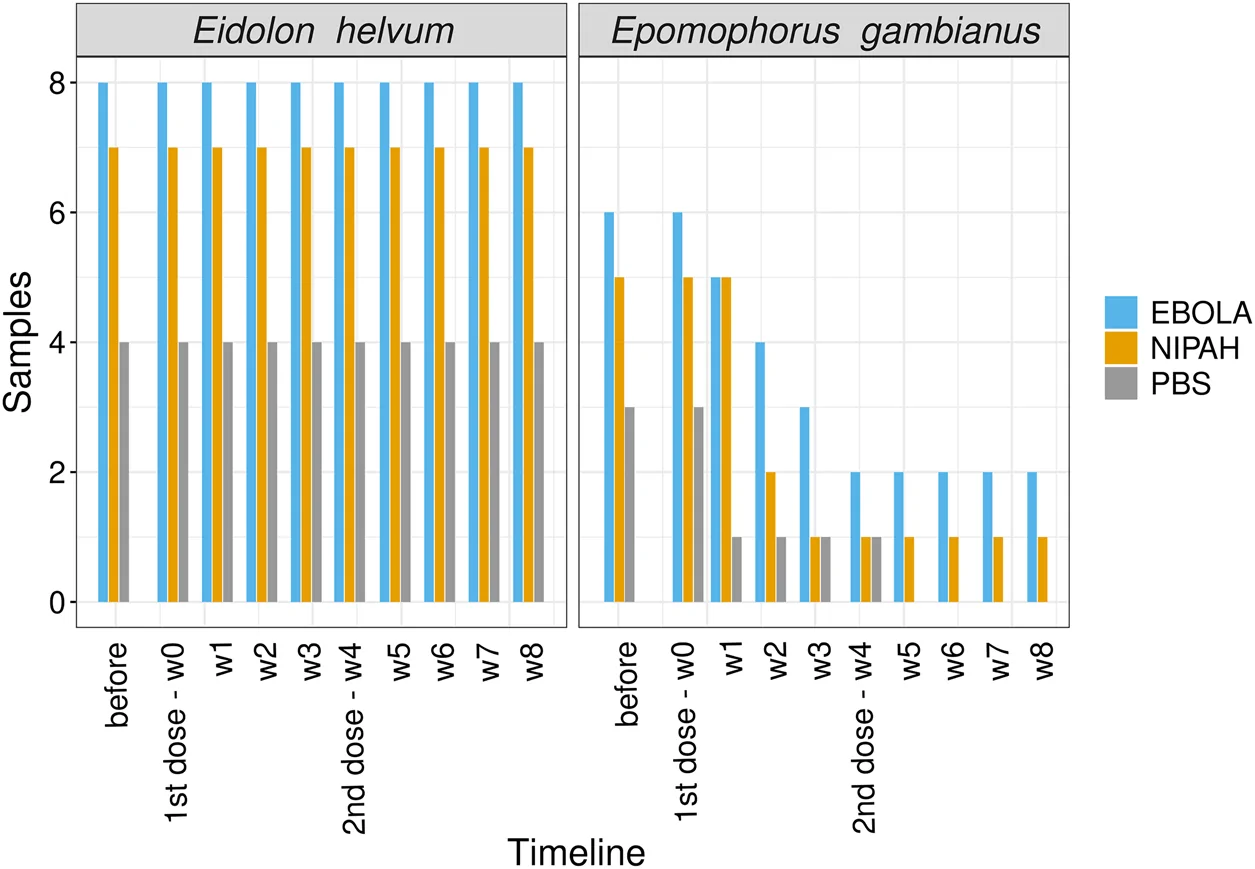
Timeline of serum sample collection by species and by experimental group. The two injections were given four weeks apart, with the ChAdOx1 vaccine given at week 0 and the MVA vaccine given at week 4. The attrition in *Ep. gambianus* numbers was owing to deaths occurring in all three groups. w = Weeks after first injection.

Serum samples were used to detect antibodies against a panel of eight viral antigens from three henipaviruses and two filoviruses (including the Ebola virus GP protein and Nipah virus G protein encoded in the vaccines) by a Luminex xMAP–based multiplex microsphere-based immunoassay (MIA) and recorded as median fluorescence intensity (MFI) units [48–51]. A subset of serum samples was also tested by pseudotyped virus neutralization assay [52]. Whole blood samples from selected time points, all collected from a representative subset of *Ei. helvum* bats, were processed for RNA sequencing, and gene expression levels were compared between groups.

## 3. Results

### 3.1. Antibody response

#### 3.1.1. Nipah vaccine

Using a Luminex MIA, we observed increases in anti-Nipah-G antibody binding in all but one *Ei. helvum* bat from the Nipah vaccine group (figure 2A). When pooling the Ebola vaccine group and the PBS group to act as a control against the Nipah vaccine group, the net change in anti-Nipah-G binding from first to last sample was significantly greater in the Nipah vaccine group (Wilcoxon rank-sum test: *W* = 76, *p* = 0.0026, figure 2C). The range of MFI values in the Nipah vaccine group at the final time point was similar to those obtained with medium-to-high concentrations of the anti-Nipah-G monoclonal antibody 102.4 (supplementary material, figure S1). The MFI increase was already significant before the second injection (median increase of 1.0 log10 unit between the first sample and the last sample before the second injection in the Nipah group, −0.5 in the Ebola group and 0.0 in the PBS group), again confirmed statistically with a Wilcoxon test by pooling the Ebola and PBS groups as a control (*W* = 77, *p* = 0.0018, figure 2B). A linear mixed-effects model applied to the whole time series of log-transformed MFI against Nipah G (with vaccine group and time as fixed variables and bat identifier as a random variable) confirmed the significant effect of the Nipah vaccine (interaction between group and time: *p* < 10^−4^, see supplementary material, text S1). We also noted a negative correlation between the net change in anti-Nipah-G antibodies and their levels on the first day (figure 3A), whether across all bats (Pearson's *r* = −0.65, 95% CI [−0.85, −0.28]) or within the Nipah vaccine group (*r* = −0.90, 95% CI [−0.98, −0.44]). One bat in the PBS group showed a large increase in anti-Nipah-G binding at the last time point, possibly following exposure to a naturally occurring paramyxovirus shed by another bat in the same cage (figure 2A).

**Figure 2.**
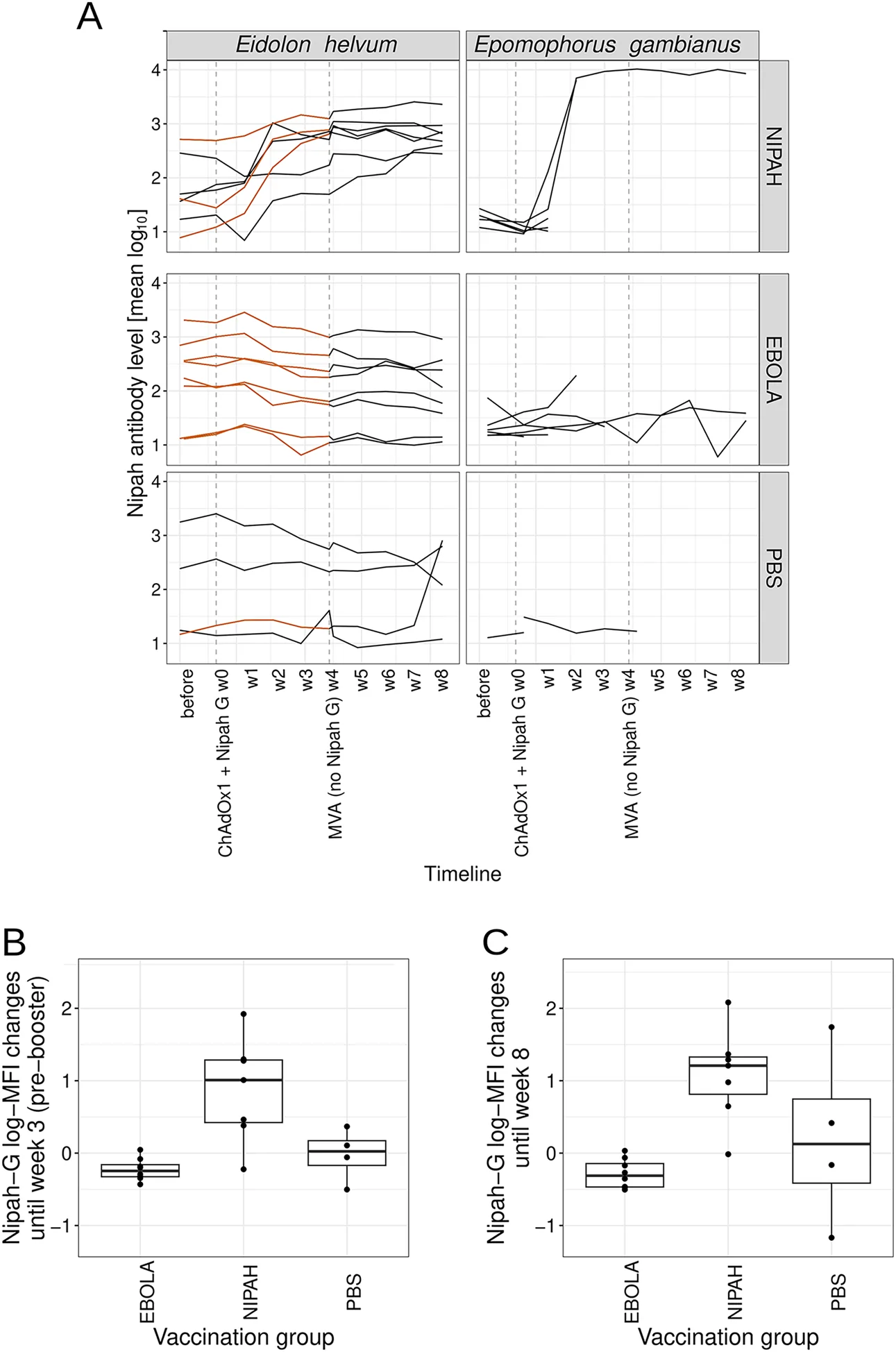
Anti-Nipah-G antibody response in all treatment groups. (A) Time series of mean log10 (MFI) values against Nipah G, by species and experimental group. As no change in anti-Nipah-G antibodies was expected in the Ebola vaccine group, this group is considered a control in this figure. Each line represents one individual bat. Vertical dotted lines show the time of injections in each group. Samples and time points for which transcriptomic data were included in the differential gene expression analysis are shown in dark orange. (B) Box plot showing the log change in Nipah-G MFI values between the initial and third week (pre-booster) for each *Eidolon helvum* treatment group. Points represent the net change across that period for each *Ei. helvum* bat. (C) Box plot showing the log change in Nipah-G MFI values between the initial and eighth (final) week for each *Ei. helvum* treatment group. Points represent the net change across that period for each *Ei. helvum* bat.

**Figure 3.**
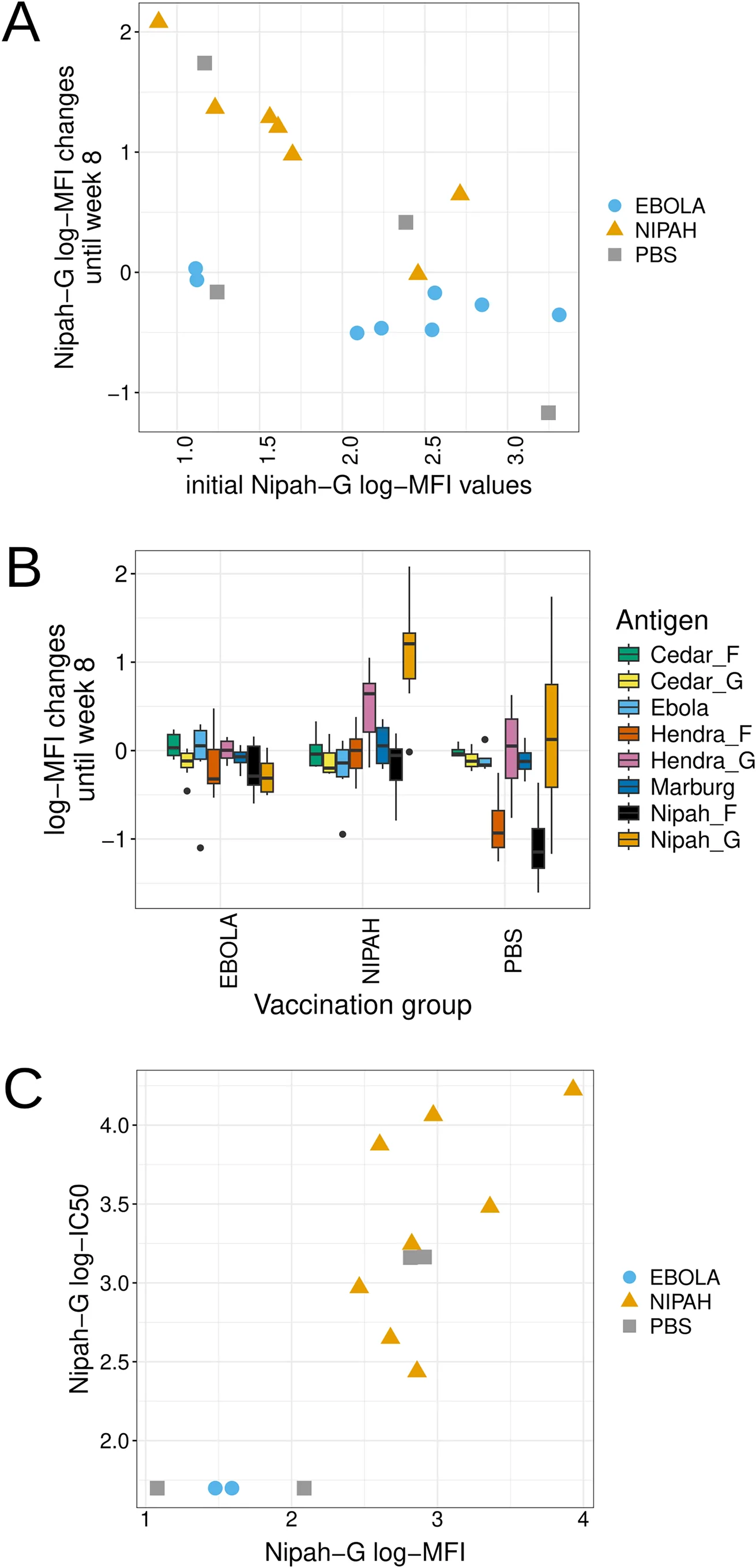
Serological response dynamics and correlations in *Ei. helvum* bats following Nipah-G vaccine delivery. (A) Association between the initial Nipah-G log-MFI value and the change in log-MFI values across the entire study period. Each dot represents a single *Ei. helvum* bat. (B) Box plot showing the changes in log-MFI values for all tested antigens across the entire study period for *Ei. helvum*. (C) Correlation between the MFI values from the Luminex-based multiplex microsphere immunoassay (MFI values against the Nipah-G antigen) and half maximal inhibitory concentration (IC50) values from the lentivirus pseudotyped virus neutralization assay (serum dilution required to achieve 50% neutralization). The starting dilution was 50-fold; four sera with an IC50 below 50-fold are shown as points with a value of 50 (approx. 1.7 on the log scale).

In our MIA panel of eight viral antigens from three henipaviruses and two filoviruses, the only other one to show a significant MFI increase in the Nipah vaccine group was the Hendra virus G protein, albeit to a lower extent than the Nipah virus G protein (figure 3B and supplementary material, figure S2).

To test whether the bats produced neutralizing antibodies after vaccine exposure, a subset of 14 serum samples, collected at the end of the experiment and selected to cover the whole range of MFI values against the Nipah G antigen, were further tested by lentivirus pseudotyped virus neutralization assay [52] (supplementary material, text S2). There was a strong positive correlation between the measurements from the two assays (Spearman's *ρ* = 0.82, *p* = 0.0003, *n* = 14), indicating the presence of neutralizing antibodies in serum samples with MFI values above 200 units (figure 3C). Although we did not analyse changes in neutralizing antibodies across the experiment, we note that all eight bats in the Nipah vaccine group had detectable neutralizing antibodies by the end of the study (figure 3C).

This pseudotype neutralizing threshold around 200 MFI units (2.3 log10 units) is slightly lower than the Nipah virus neutralization cut-off value of 500 MFI units (2.70 log10 units) previously established for Nipah G MIA in *Ei. helvum* [53]. The fact that several *Ei. helvum* bats in our study had initial MFI values above these thresholds (four above 500 MFI units and eight above 200; supplementary material, figure S3) is consistent with previous reports, for example 46% of wild-caught adult *Ei. helvum* from Ghana with values above 500 [36]. In the Nipah vaccine group, six out of seven *Eidolon* bats were initially below 500, and only two were still below this at the end of the experiment (figure 2).

In contrast, none of the *Epomophorus* bats in the study had initial anti-Nipah-G binding levels above 500 MFI units. The two *Epomophorus* bats that survived for more than a week post prime injection showed a more pronounced pattern of seroconversion within days (figure 2A).

#### 3.1.2. Ebola vaccine

In sharp contrast to the response to the Nipah vaccine, there was no consistent increase in anti-Ebola antibodies in the *Eidolon* bats that received the Ebola vaccines (figure 4). The four *Epomophorus* bats that survived for at least a week post prime Ebola vaccine injection did, however, show large increases in anti-Ebola glycoprotein MFI values. The response to the Ebola vaccine in this species was similar in magnitude to the range of MFI values against serial dilutions of the anti-Ebola glycoprotein monoclonal antibody 5D2 (supplementary material, figure S1).

**Figure 4.**
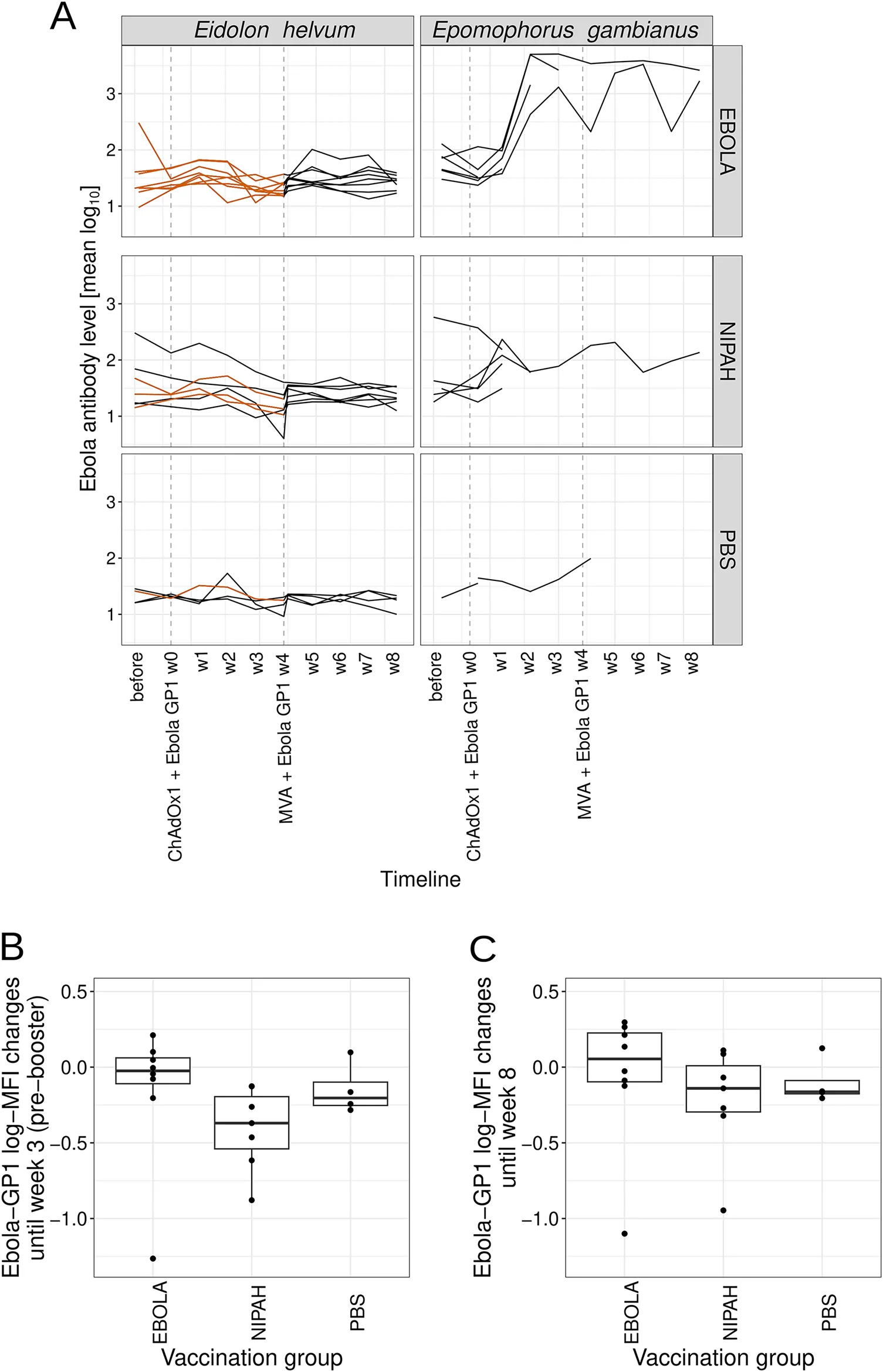
Anti-Ebola-GP1 antibody response in all treatment groups. (A) Time series of mean log10 (MFI) values against Ebola, by species and experimental group. As no change in anti-Ebola-GP1 antibodies was expected in the Nipah vaccine group, this group is considered a control in this figure. Vertical dotted lines show the dates of injections in each group. Samples and time points for which transcriptomic data were included in the differential gene expression analysis are shown in dark orange. (B) Box plot showing the log change in Ebola-GP1 MFI values between the initial and third week (pre-booster) for each *Ei. helvum* treatment group. Points represent the net change across that period for each *Ei. helvum* bat. (C) Box plot showing the log change in Ebola-GP1 MFI values between the initial and eighth (final) week for each *Ei. helvum* treatment group. Points represent the net change across that period for each *Ei. helvum* bat.

We tested 15 serum samples collected on the last day of the experiment using our lentivirus pseudotype neutralization assay (expressing the Ebola virus GP protein [54]), comprising eight *Eidolon* bats from the Ebola vaccine group and four from the PBS group, as well as three *Epomophorus* bats that completed the study (two from the Ebola vaccine group and one from the Nipah vaccine group) (supplementary material, text S2). None of the samples neutralized the Ebola pseudotype virus at the lowest dilution (1 : 50).

The heavily glycosylated mucin domain within the Ebola virus GP1 protein has been shown to inhibit antibody binding protein [55]. To investigate whether this was causing the discrepancy between the two assays for the *Ep. gambianus* sera, we repeated the pseudotyped virus neutralization assay after deleting the mucin domain from the Ebola virus GP1 sequence. However, once again, none of the bat serum samples neutralized the pseudotype virus at the lowest dilution (1 : 50).

As a further control using an *in vitro* fluorescence infection assay, we confirmed that both vaccine vectors were able to infect *Ei. helvum* and *Ep. gambianus* cells with similar efficiency to known permissive human cells (supplementary material, text S3 and figure S4).

### 3.2. Differential expression of genes in vaccine-exposed *Eidolon helvum*

To identify changes in gene expression levels in the vaccine recipients, we analysed the transcriptome from whole blood samples collected until four weeks after prime vaccine delivery (before the booster was given) from a subset of *Ei. helvum* bats. No transcriptomic analysis was conducted for *Ep. gambianus*. After excluding samples with low-quality RNA following extraction, as well as one bat from the PBS-injected group that had high antibody levels against the Nipah G protein at capture (supplementary material, figure S5), we carried out sequence analysis using data from one PBS individual (which had consistently low antibody levels against all tested antigens for at least seven weeks), two or three Nipah vaccine–treated bats and six to eight Ebola vaccine–treated bats depending on the time point (figure 5A, supplementary material, table S1). While the PBS-injected bat used as control here eventually seroconverted at week 8, this is unlikely to have affected its gene expression on week 4 or earlier. Given the low numbers of available bats, the pairwise comparisons between the PBS and vaccine groups should be considered preliminary and serve as a foundation for future studies. The reads were mapped to an in-house-generated *Ei. helvum* draft genome assembly and annotation (available on figshare [57]), which was generated using published data from Parker *et al.* [58] as well as newly generated mate-pair next-generation sequencing data. Since a large proportion of the blood transcripts (approx. 75%) mapped to haemoglobin, it is possible that lowly expressed genes might not have been recovered in the blood samples. In the following, we present selected results from two sets of comparisons, focusing on genes and pathways with known immunological relevance (see supplementary material for further results).

**Figure 5.**
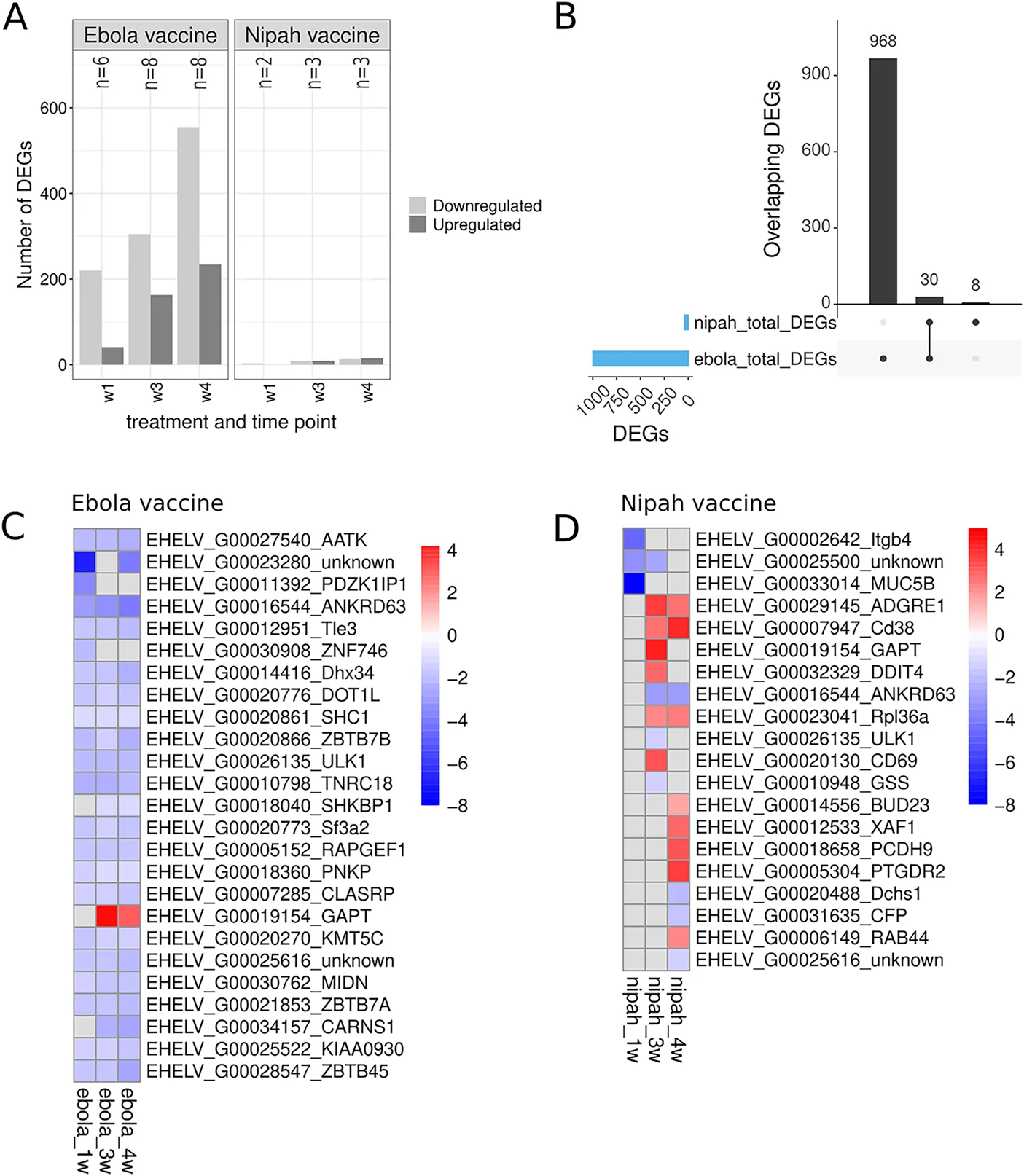
Differentially expressed genes in the *Ei. helvum* vaccine recipients. (A) Number of differentially expressed genes for the Ebola and Nipah vaccine treatment groups for each time point (false discovery rate (FDR) < 0.05) when compared with the PBS individual. 1–4w: Blood samples collected weekly. Sample size for each vaccine group is displayed above the barplot. The PBS group had one sample per time point. (B) UpSet plot of the intersection of differentially expressed genes (DEGs) in the Ebola and Nipah vaccine treatment groups. The nature of a given intersection is indicated by the dots below the barplot. For instance, the genes (*n* = 968) in the first column were differentially expressed in the bats retrieving the Ebola vaccine but not the Nipah vaccine. (C and D) Heat map illustrating the log2 fold change of the top 10 DEGs (rows) from each time point in the (C) Ebola and (D) Nipah vaccine groups. Red shading indicates that gene expression was higher, and blue shading indicates that gene expression was lower in the vaccine group than in the PBS group. Grey colour indicates time points when the genes were not significantly differentially expressed. Genes that could not be assigned a gene name from the in-house-generated *Ei. helvum* draft genome are labelled unknown. All DEGs from the vaccine groups are listed in supplementary material, datasets S1–S2 [66].

#### 3.2.1. Pairwise comparisons between the phosphate-buffered saline group and each vaccine group in *Eidolon helvum*

The number of differentially expressed genes (DEGs) ranged from 261 to 789 (mean ± s.d., 506 ± 266.0) and 3 to 28 (16.3 ± 12.6) when conducting a pairwise comparison for every time point between the PBS individual and the Ebola or Nipah vaccine group, respectively (figure 5A). Of the 38 genes differentially expressed at least once in the Nipah vaccine group, 30 were also differentially expressed at least once in the Ebola vaccine group (figure 5B and supplementary material, figure S6). Some genes with function in immunity were among the top DEGs in both vaccine groups (figure 5C,D), including *GRB2 Binding Adaptor Protein Transmembrane* (*GAPT*), which is predicted to be involved in B cell homeostasis and B cell proliferation [59]. However, the vaccines also elicited specific responses with 968 and eight uniquely DEGs in the Ebola and Nipah vaccine groups, respectively (figure 5B and supplementary material, figures S7–S9). Out of the eight genes uniquely differentially expressed in the Nipah vaccine group (figure 5B and supplementary material, figure S6), some are linked to immune-related processes, such as inflammatory signalling (*C1q and tumour necrosis factor-related protein 6*, *C1QTNF6*, [60]), mucosal barrier function (*mucin 5B, oligomeric mucus/gel-forming*, *MUC5B*, [61]) and regulation of haematopoietic cell differentiation (*growth factor independence 1B*, *GFI1B*, [62]).

We performed a gene ontology (GO) [56] analysis to investigate whether certain Kyoto Encyclopedia of Genes and Genomes (KEGG, [63]) pathways or biological processes were over-represented among DEGs in the vaccine-exposed *Ei. helvum* bats. Bats in the Nipah vaccine group had no over-represented KEGG pathways or biological processes. In contrast, several GO terms involved in leukocyte differentiation were over-represented in the Ebola vaccine-exposed bats when compared with the PBS group (supplementary material, figures S10 and S11). These included the T-helper (Th)1 and Th2 cell differentiation pathways, as well as the Notch signalling pathways and the Janus kinase (JAK)-signal transducer and activator of transcription (STAT) cascade which are both involved in Th1 or Th2 cell differentiation. The fold change of some of the significant DEGs from the Th1 and Th2 cell differentiation pathway in Ebola vaccine-exposed bats is visualized in figure 6 and discussed in more depth in text S4 and figure S12 in the supplementary material. It is important to note that the immune response we assessed was triggered by a vaccine vector carrying the viral glycoproteins, rather than by exposure to live viruses.

**Figure 6.**
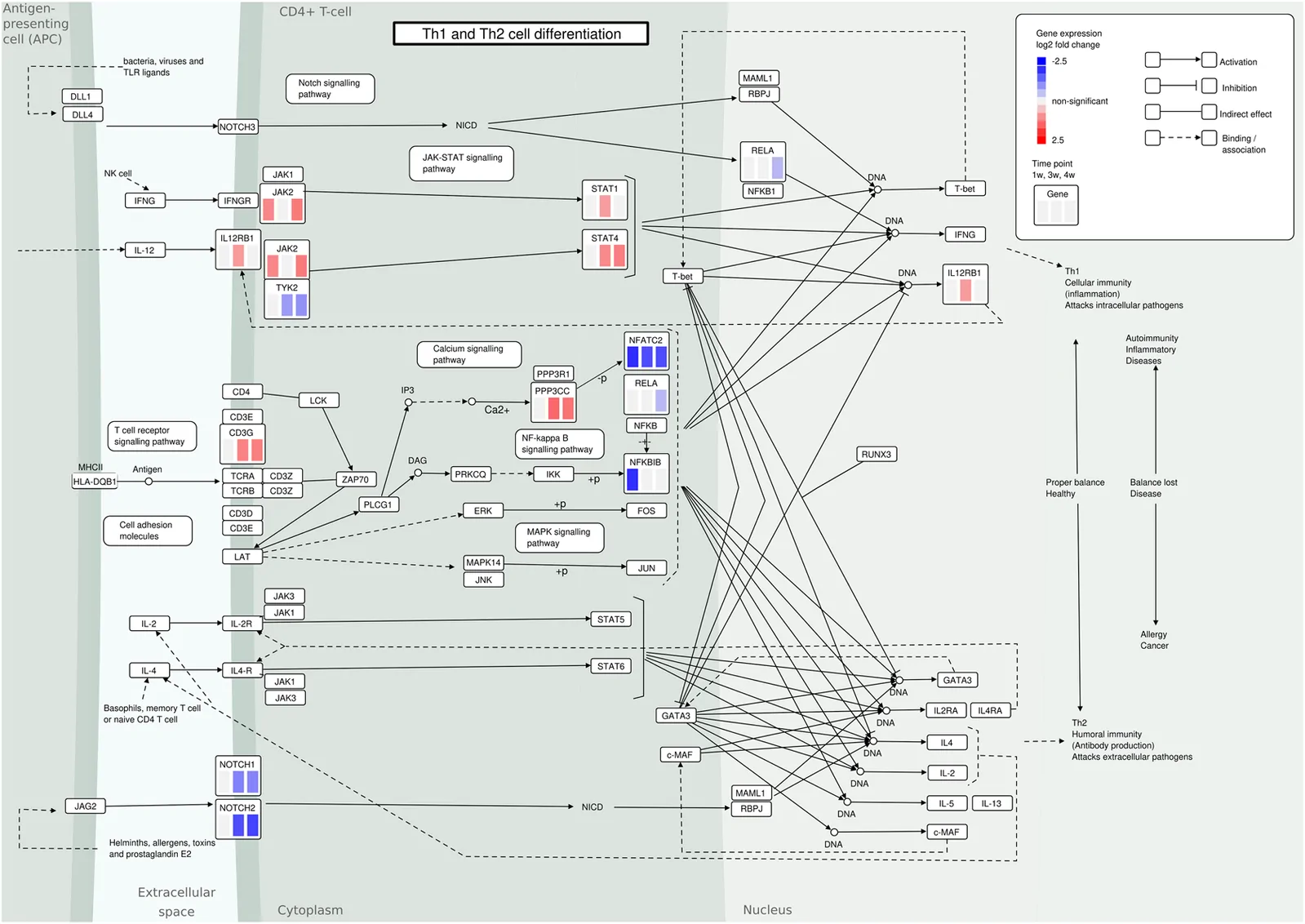
Log2 fold changes of the expression level between the *Ei. helvum* Ebola vaccine and PBS group mapped on the Th1 and Th2 cell differentiation pathway (GO:hsa04658) from the KEGG database [63–65]. Red shading indicates that gene expression was significantly higher, and blue shading indicates that gene expression was significantly lower in the Ebola vaccine group than in the PBS group (*n*_PBS_ = 1, *n*_Ebola_ = 6–8; for exact number of individuals, see figure 5A). Genes not significantly differentially expressed are shown in grey for that particular time point. Each box represents one gene in the pathway, and the columns within the box show the gene expression fold change for blood samples at the different time points; 1, 3, and 4w post prime vaccine, from left to right. Data are shown for genes that were differentially expressed at least one time point. w = weeks.

#### 3.2.2. Pairwise comparisons between the Ebola and Nipah vaccine groups

We further conducted a direct pairwise differential gene expression analysis between the *Ei. helvum* Ebola and Nipah vaccine groups to identify genes that were differentially regulated in the two vaccine groups during the first four weeks post prime vaccine exposure. The numbers of DEGs for each of those four weeks ranged from 185 to 788 (405 ± 282), with the highest number of DEGs occurring at one week and four weeks post prime vaccine exposure (supplementary material, table S2 and dataset S3 [66]). Although the immune response we observed was induced by a vaccine vector carrying viral glycoproteins rather than live viruses, several GO terms commonly associated with responses to authentic viral infections were differentially regulated when comparing the two vaccine groups. Notably, GO terms involved in type I interferon response, response to virus and T-cell selection were over-represented in the DEGs between the two vaccine groups (figure 7 and supplementary material, figures S13 and S14). Several interferon response genes that were differentially expressed between the two vaccine groups were downregulated in the Ebola vaccine group but upregulated in the Nipah vaccine group when compared with the PBS group (i.e. IFIT1-2 and IRF7, figure 7B).

**Figure 7.**
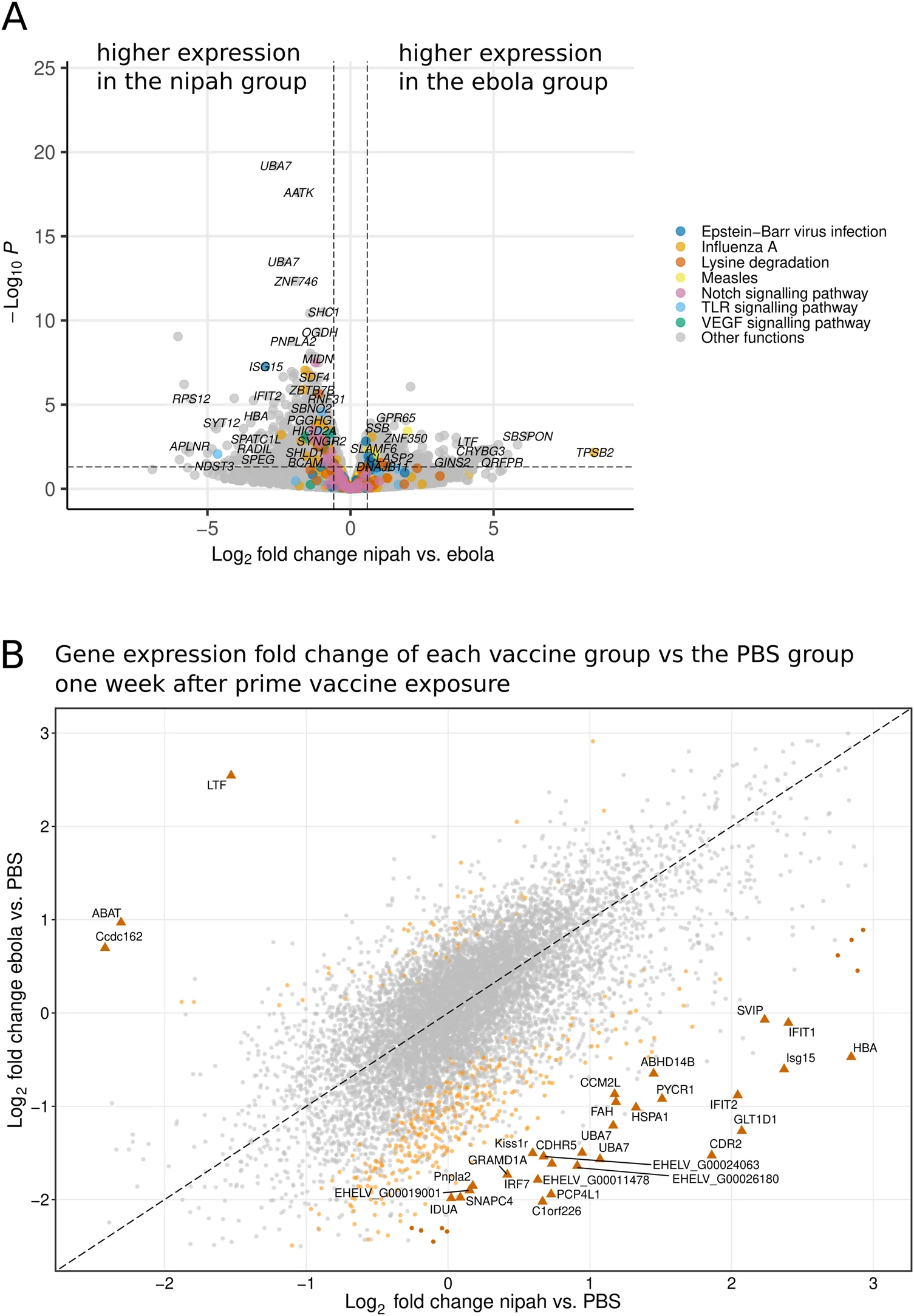
(A) Volcano plot displaying log2 fold change in gene expression between the *Ei. helvum* Nipah and Ebola vaccine groups versus log10 transformed *p*-value one week after prime vaccine exposure. Genes from over-represented KEGG pathways [63] (supplementary material, figure S12) are visualized in colour (*n*_Ebola_ = 6–8, *n*_Nipah_ = 2–3; for exact number of individuals, see figure 5A). (B) Log2 fold change of genes from each vaccine group compared with the PBS group. Orange dots denote significantly differentially expressed genes between the Nipah and Ebola vaccine groups, dark orange dots denote significantly differentially expressed genes with a log2 fold change of at least 2, and triangles denote differentially expressed genes upregulated in one vaccine group and downregulated in the other when compared with the PBS group (*n*_Ebola_ = 6–8, *n*_Nipah_ = 2–3, *n*_control_ = 1; for exact number of individuals, see figure 5A). Data from other time points are shown in the supplementary material, figures S15–S17.

The top DEG between the Ebola and Nipah vaccine groups one week after prime vaccine exposure was ubiquitin-like modifier-activating enzyme 7 (UBA7) (figure 7 and supplementary material, figure S18). UBA7 encodes a member of the E1 ubiquitin-activating enzyme family and is involved in interferon-stimulated gene 15 (ISG15) protein conjugation, a process known as ISGylation [67]. Interestingly, *ISG15* as well as other key players in the ISGylation pathway (i.e. *HERC5* and *USP18*) were also differentially expressed between the Nipah and Ebola vaccine group one week after prime vaccine exposure. The fold change of significant DEGs from the ISGylation pathway, when comparing the two vaccine groups one week after the initial vaccine exposure, is visualized in figure 8. These four genes all had lower expression in the Ebola vaccine group than in the Nipah vaccine group. When compared with the PBS group, only *UBA7* was significantly downregulated at this time point in the Ebola vaccine group (supplementary material, dataset S2 [66]). While ISGylation is commonly associated with antiviral immune responses induced by type I interferon, which plays a key role in inhibiting viral replication and defending host cells against viral infections [67], the upregulation of this pathway in our study reflects immune activation in response to the ChAdOx1 vaccine vector and the Nipah-G and Ebola GP1 proteins.

**Figure 8.**
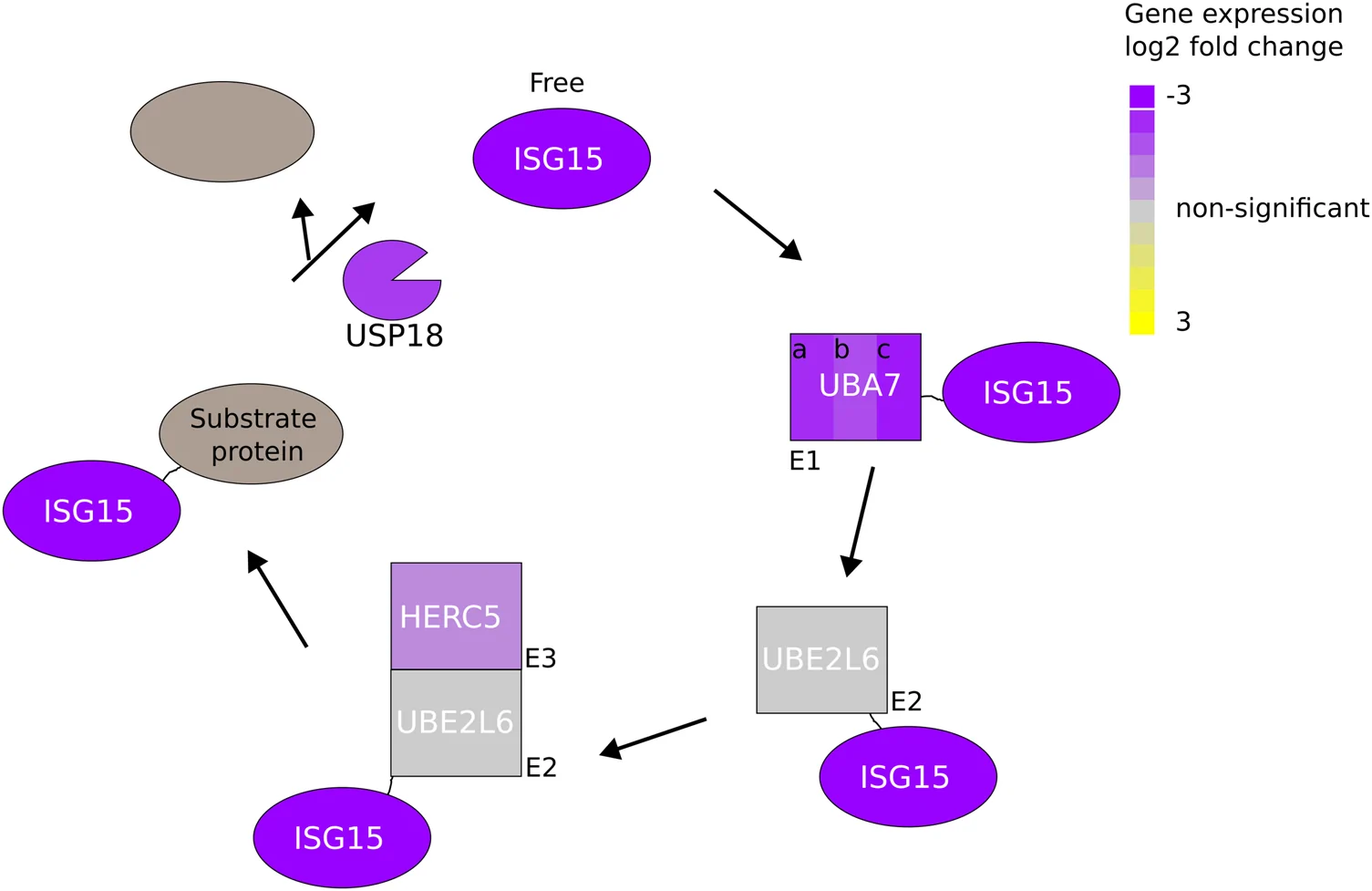
Differential gene expression between the *Ei. helvum* Ebola and Nipah vaccine groups one week after initial vaccine exposure mapped to the ISGylation pathway. Log2 fold changes in gene expression are displayed for genes involved in the ISGylation signalling pathway (*n*_Ebola_ = 6–8, *n*_Nipah_ = 2–3; for exact number of individuals, see figure 5A). As described in the literature, ISG15 is attached to substrates in a three-step enzymatic (E) cascade. First, it is activated by E1 UBA7, then it is transferred to the E2 UBE2L6 and subsequently to a target protein, such as a viral protein, through the E3 HERC5 [67]. Purple shading indicates significantly lower gene expression in the Ebola vaccine group than in the Nipah vaccine group, while genes not significantly differentially expressed (*UBE2L6*) are shown in grey. *UBA7* has three different shadings as reads mapped to three different locations on our in-house-generated *Eidolon helvum* genome assembly, all annotated as UBA7. UBA7a = EHELV G00034959, UBA7b = EHELV_G00003507 and UBA7c = EHELV_G00003508.

Further DEGs and over-represented GO terms with known immunological relevance are presented in text S5 in the supplementary material.

## 4. Discussion

### 4.1. Serological response to Nipah vaccine

Serum antibody binding to bat henipavirus antigens using the Luminex MIA platform was previously validated *in vitro* against neutralization assays using Hendra and Nipah viruses [53]. While indicative of the protective function of antibodies in bats, it does not allow inference on the history of viral exposure or the kinetics of antibody production. Studying antibody kinetics in controlled experiments is key for interpreting wildlife serological data [68] but is challenging owing to the need of containment level 4 facilities. In this study, we measured antibody responses to Nipah and Ebola antigens in *Ei. helvum* and *Ep. gambianus* in an experimental setting to obtain positive controls for antibody levels in bats.

MFI values observed in *Ei. helvum* after Nipah vaccine injection are comparable to those found in wild *Ei. helvum* populations [5,43,69] and to values obtained against monoclonal antibodies (supplementary material, figure S1). Furthermore, the steep increase in anti-Nipah-G antibody levels mirrors patterns observed in a captive colony of *Ei. helvum* bats in Ghana [70] with ongoing henipavirus circulation [71,72], where MFI increased by several thousand units within a three-month period [70]. Similar seroconversion was previously reported in *Pteropus* bats within two to four weeks after henipavirus infection [14,32]. While few studies have successfully detected paramyxovirus RNA or antibodies in *Ep. gambianus* (generally reporting lower prevalence than in *Ei. helvum* [38,43,44]), we observed a very strong antibody response in the few bats of this species that we were able to monitor.

An important caveat is that, in the absence of a reliable antigen measurement assay for individual bats (owing to the low sensitivity of PCR on non-lethal bat samples [73]), we could not test bats for active henipavirus infection. The bats had unknown, probably diverse infection histories, as illustrated by their diverse pre-vaccine antibody levels. Thus, some of the observed seroconversions may have resulted from close-contact transmission of pre-existing viruses within the cages, such as the seroconversion recorded on the last time point in one individual from the PBS group. However, we previously found that such natural seroconversion events were rare in captive *Ei. helvum*, with a mean waiting time to seroconversion of 2.5 years for immunologically naive bats in a 100-strong captive colony [70].

This study was not designed to measure antibody waning, but one *Ei. helvum* individual in the PBS group showed a steady decline in antibody levels over the eight weeks of sampling. Whereas previous studies inferred antibody decay in *Ei. helvum* over months or years, either indirectly from wild-caught bats of known age [69] or directly from longitudinal captive studies [70], this is the first study to document rapid decay on a weekly basis.

### 4.2. Species-specific differences in the antibody response against the Ebola vaccine

The absence of detectable antibody response to the Ebola vaccine in *Ei. helvum* was unexpected and contrasts with the strong response to the same vaccine in *Ep. gambianus*. This discrepancy is notable because both species mounted a clear antibody response to a Nipah virus vaccine that used the same adenovirus vector. This vector does not display viral antigens directly but encodes the glycoprotein gene from the Ebola or Nipah virus, respectively, requiring host cell expression to induce an immune response.

Although some studies have detected antibodies against Ebola viruses in a small number of wild-caught *Ei. helvum* [17,40], there is compelling experimental evidence that a mutation in the filovirus receptor NPC1 unique to this species of bats inhibits cell entry by Ebola virus *in vitro* [29]. However, we used an adenovirus vector which targets the coxsackie and adenovirus receptor (CAR), and we confirmed that the Ebola virus (EBOV) vaccine is able to enter *Ei. helvum* cells *in vitro* (supplementary material, figure S4). Another possible mechanism for the observed differences in antibody response to the Nipah and Ebola vaccines in *Ei. helvum* is the varying ability of tetherin (Bst2) to inhibit different viruses. Tetherin blocks viral particle release by linking them to the host cell membrane [74] and plays a key role in bat antiviral responses [75]. Interestingly, one study showed that tetherin inhibits Ebola virus replication [76], while another found that it inhibits Nipah but not Ebola virus replication [77] in Buettikofer's epauletted fruit bat (*Epomops buettikoferi*) cells. In our analysis, however, there was no evidence of differential expression of the tetherin gene *Bst2*.

For the Nipah vaccine, MIA and a pseudotyped virus neutralization assay showed concordant results. A similar agreement (albeit with negative results only) was observed for the Ebola vaccine in *Ei. helvum*. In contrast, we noted a discordant antibody result for *Ep. gambianus* vaccinated against Ebola: the MIA detected high MFI signal for antibody binding to the Ebola GP, yet these antibodies failed to neutralize a corresponding pseudotyped virus. Similar discordances between Ebola virus GP binding antibodies and virus neutralization titres have been made in experimental vaccination studies in animals and people as well as studies from naturally infected human survivors [78,79]. Our own results suggest that *Ep. gambianus* may produce GP-specific antibodies that are reactive to non-neutralizing epitopes on the protein. While non-neutralizing antibodies may not directly block viral entry, they can still play a role in immune response through Fc-mediated effector functions, such as phagocytosis or natural killer (NK) cell activation [80]. In the context of Ebola virus, both neutralizing and non-neutralizing antibodies have been associated with protection from infection [81]. However, it is important to note that our understanding of the kinetics and function of antibodies during and after viral infection in bats is limited. Further studies are needed to assess whether the antibodies detected in *Ep. gambianus* provide protection against Ebola virus.

We acknowledge that the statistical power of this study was limited by the small number of animals, especially *Ep. gambianus*, where the few surviving individuals may not be representative of the species as a whole. Additionally, the unavailability of positive control serum from bats with known Ebola virus infection remains a significant challenge for assay validation. Future technical work should focus on optimizing serological assays, for instance by investigating the impact of the Ebola GP mucin-like domain on antigen presentation, to better estimate the sensitivity and specificity of these tools in bats.

### 4.3. Differential gene expression

#### 4.3.1. Gene expression response in the Ebola vaccine group

By comparing the gene expression profiles between the *Ei. helvum* Ebola and Nipah vaccine groups, we aimed to identify potential differences that could explain the observed variation in antibody production. One key finding when comparing each vaccine group to the control individual was that several GO terms related to Th cell differentiation were over-represented in the Ebola vaccine group, while this was not observed in the Nipah vaccine group. Th cells play a crucial role in immune function, differentiating into Th1 or Th2 subtypes, each promoting different immune responses. Th1 differentiation, typically driven by intracellular pathogens, is associated with IFN-γ and IL-12 secretion [82]. In contrast, Th2 cells, typically stimulated by extracellular antigens, help initiate the humoral immune response by activating naive B cells to secrete IgM and other antibodies such as IgA, IgE and IgG [82]. Owing to limited detection of cytokine transcripts, we were unable to determine which Th cell subtypes predominated in the Ebola vaccine group. As a result, we cannot conclude whether a potential shift towards cellular immunity, rather than humoral immunity, contributed to the lack of antibody production in Ebola-vaccinated *Ei. helvum* bats. Further studies are needed to better understand the regulation of Th cell differentiation in bats following immune challenge and viral infection.

While drawing direct comparisons between our results and responses to real infections is challenging, one over-represented GO term in our study (the JAK/STAT pathway) was also enriched in a study comparing Ebola and Marburg virus responses in human and *R. aegyptiacus* bat cell lines [83], suggesting some overlap between vaccine-induced and infection-induced responses.

#### 4.3.2. Gene expression response in the Nipah vaccine group

Despite the increase in antibodies against the Nipah G protein in the bats following Nipah vaccine exposure, few genes were differentially expressed and no GO terms were over-represented in the Nipah vaccine group. It is important to note that fewer Nipah vaccine-exposed individuals were available for analysis than Ebola vaccine-exposed individuals, and that additional samples might have revealed more patterns.

#### 4.3.3. Pairwise comparisons between the Ebola and Nipah vaccine groups

Since changes in gene expression to each vaccine group probably reflect responses to the vaccine vector and the G proteins, we also directly compared the vaccine groups. As both groups received the same vector with either the Ebola or Nipah glycoprotein during the prime injection, the observed differences between the two groups are probably attributable to these distinct glycoproteins. Pairwise comparisons showed that key players in the ISGylation pathway had lower expression in the Ebola vaccine group than in the Nipah vaccine group shortly after prime vaccine exposure. While ISGylation and type I interferon responses are typically associated with viral restriction through the modification of host/viral proteins to inhibit replication or the release of viruses [67,84], the vaccines may have engaged innate immune sensing pathways via pattern recognition receptors (PRRs), thereby triggering this antiviral state in the absence of viral replication.

When looking at gene expression patterns of the ISGylation pathway in filovirus infections in bats, several genes (*ISG15*, *USP18*, *HERC5* and *UBE2L6*) were significantly upregulated in the blood of Marburg virus-infected Egyptian fruit bats [85]. In another study, genes involved in ISG15-ylation were upregulated in Egyptian fruit bat cells infected with a recombinant Marburg virus carrying mutations in the IFN inhibitory domain region of the viral protein 35 (VP35), but not in bat cells infected with wild-type Marburg virus [86]. While these findings are derived from viral infections, they may help contextualize the potential role of ISGylation in immune responses to viral components in the vaccine model we used.

### 4.4. Conclusion

This study provides rare insight into the *in vivo* immune responses in two species of African fruit bat following exposure to Ebola or Nipah virus antigens. Remarkably, one bat species (*Ep. gambianus*) developed detectable antibodies against both virus antigens, while the other bat species (*Ei. helvum*) only developed antibodies against Nipah virus antigens. A simultaneous transcriptome analysis in *Ei. helvum* revealed that few antiviral genes and pathways were differentially expressed in response to the Nipah vaccine, while genes involved in ISGylation and T-cell differentiation pathways were differentially expressed in response to the Ebola vaccine. We acknowledge important limitations in our study design, notably the absence of female bats (to minimize complications caused by pregnancy) and the rapid depletion of *Epomophorus* bats (potentially introducing a survivorship bias), which will have to be addressed in future studies. Our findings, while preliminary, highlight immune mechanisms that might explain the specificity of host–virus associations in bats.

## 5. Material and methods

### 5.1. Facility and animals

Wild bats were caught at their roosts in Accra, Ghana, in June 2017, using mist nets. We targeted two species of fruit bat, the African straw-coloured fruit bat *Ei. helvum* and the Gambian epauletted fruit bat *Ep. gambianus*. Any juvenile or adult female bats captured were released immediately, while adult males were placed in individual canvas bags and transported by road to the nearby purpose-built experimental facilities maintained by the Wildlife Division of the Government of Ghana in Achimota Forest, Accra. A total of 75 bats were captured over three consecutive nights. We only kept adult males (27 *Ei. helvum* and 33 *Ep. gambianus*) to reduce physiological variability owing to age or pregnancy status, and the number of animals was constrained by logistical and ethical factors. After an initial observation period to exclude unhealthy or aggressive bats, 19 *Ei. helvum* and 14 *Ep. gambianus* bats were individually tagged with intradermal microchips and were placed in smaller cages (80 × 80 × 80 cm) in single-species groups of up to five bats for the rest of the study (two months). These cages were suspended from the roof of the animal house and separated by approximately 2 m of space and tin baffles to prevent direct physical contact between bats and indirect contact via droplets (urine, secretions, food and water). Bats received diced mixed fruits (e.g. mango, papaya and banana) and ad libitum water, which were replaced every day [87]. Bats were monitored by veterinarians for 8–10 days before the start of the experiment. During the experiment, bats were regularly assessed by veterinarians for signs of distress, weight loss or injury. If the welfare of any bat was seriously reduced, it was euthanized on humane grounds. Unexpectedly, *Epomophorus* bats did not cope well with captivity and either died suddenly or had to be euthanized on the basis of weight loss: four survived until the second injection and only three completed the whole two-month study (figure 1).

### 5.2. Vaccines

Four live recombinant viral vaccines were produced by the Viral Vector Core Facility (University of Oxford) for the purpose of this study. Two were recombinant chimpanzee adenovirus (ChAdOx1), one of which encoded the Zaire ebola virus (*Orthoebolavirus zairense* Makona strain) GP protein and the other a Nipah virus (*Henipavirus nipahense* Bangladesh strain) G protein. The other two vaccines were MVA, one encoding the same Zaire ebolavirus Makona G protein and the other with no added genes following unsuccessful attempts to insert the Nipah virus G protein into the MVA. This heterologous ‘prime-boost’ vaccination strategy (adenovirus vector followed by boosting with MVA vector) was previously validated in clinical trials of Ebola vaccines [47] and in animal experiments against diverse pathogens [88,89]. The presence of the antigen in each vaccine was confirmed by ID PCR. For each vaccine, the recombinant virus was titrated and assayed by spectrophotometry to measure the number of virus particles ml^−1^. The sterility of the virus was confirmed by inoculation of TSB broth with 10 µl of purified virus and incubation for 3 days at 35°C. See results of quality control in the supplementary material (viral vectors). The vaccines were shipped on dry ice to the University of Ghana, and the cold chain was maintained until the vaccines were used.

### 5.3. Experimental design

Bats of each species were arbitrarily split into three groups for injections with the Ebola vaccines, the Nipah vaccines or PBS, respectively. Given a maximum capacity of 50 bats in the facilities, the original study design aimed for 25 bats from each species, to be housed in groups of five. Owing to premature release or euthanasia of captured bats on welfare grounds during the initial observation period, only 19 *Ei. helvum* (*n*_Ebola_ = 8, *n*_Nipah_ = 7, *n*_PBS_ = 4) and 14 *Ep. gambianus* (*n*_Ebola_ = 6, *n*_Nipah_ = 5, *n*_PBS_ = 3) were eventually enrolled in the study. An initial blood sample of 0.5 ml was collected from each enrolled bat prior to vaccine exposure. Bats were first injected with either the ChAdOx1 vaccine (dosed to *ca* 10^7^ IU) or PBS intramuscularly (volume 0.25 ml) and four weeks later with either the MVA vaccine (dosed to *ca* 10^7^ pfu) or PBS. Blood samples of 0.3 ml were collected from the propatagial vein of each bat weekly for up to eight weeks, starting on the day of the first injection (in *Eidolon*) and the day after the first injection (in *Epomophorus*). Finally, on week 8 after the initial injection, all bats were euthanized by overdose of intravenous ketamine/medetomidine, then exsanguinated, and samples from a range of organs (brain, liver, spleen, kidney and where possible lymph node) were harvested. Every blood sample was immediately split into two aliquots, to one of which an RNA preservative (RNAlater) was added to stabilize the RNA. Organ samples were preserved with the same RNA preservative. Stabilized whole blood, remaining whole blood and organ samples were transported on ice to the Biochemistry Department (University of Ghana). The whole blood aliquot (without RNAlater) was centrifuged, and serum was split between two tubes. Serum samples were heat-treated at 56°C for 30 min to inactivate any pathogens. All samples were stored at −80°C at the University of Ghana, and one serum aliquot as well as organ samples were shipped to the Institute of Zoology in London in a liquid nitrogen dry shipper for analysis, in accordance with export and import permits delivered by Ghana and UK government agencies, respectively.

### 5.4. Serology

#### 5.4.1. Multiplex microsphere-based immunoassay

We used a Luminex xMAP–based multiplex MIA to measure antigen-specific antibody binding, recorded as MFI units [48–51]. The Bio-Plex LX100/200, RRID:SCR_018025 (Luminex Corporation, Austin, TX) was used to measure the relative levels of bat serum antibodies against a panel of eight recombinant viral glycoprotein antigens (from three henipaviruses and two filoviruses): Cedar virus F and G proteins, Hendra virus F and G proteins, Nipah virus F and G proteins, Ebola virus GP protein (NC_002549.1) and Marburg virus GP protein, produced as native-like, soluble, oligomeric trimers (F and GP) or tetramers (G) [49,50,90,91]. As positive controls, serial dilutions of four monoclonal antibodies (5D2 against Ebola virus glycoprotein, 2B8 against Marburg virus, 102.4 against Hendra/Nipah G protein and 14F3 against Cedar virus) were tested against the same panel of antigens alongside bat serum samples (supplementary material, figure S1). As negative controls, two wells per plate were filled with the same reagents but no serum (supplementary material, figure S19). MFI values from *Ei. helvum* serum samples against the Nipah virus G protein were previously shown to correlate with neutralization assay titration against Hendra virus and Nipah virus [53] and have been used in several studies to monitor seroprevalence and infer virus circulation in *Ei. helvum* bat populations (e.g. [70,92]). Peel *et al.* [53] suggested 500 MFI as a cut-off point for Nipah virus G protein assay to maximize sensitivity based on receiver operator characteristic (ROC) curve analysis against neutralization data using serum from wild *Ei. helvum*. To date, we are not aware of similar validation data for other antigens or other bat species.

A total of 413 serum samples were tested by MIA in duplicate against the panel of eight antigens alongside negative controls. Of these, 13 readings failed and 12 samples had to be discarded after quality control as they yielded fewer than 100 microspheres. After log10 transformation of the MFI values, we flagged a small number of samples with duplicate variation above 0.5 units but did not exclude them. Here we focus on the MIA data for the Nipah G and Ebola G antigens as these matched the vaccine antigens; other antibodies showed very little temporal variations (figure 3B and supplementary material, figure S2). Because of the low number of *Ep. gambianus* bats that could be tested, we did not carry out any statistical tests on the data from this species.

#### 5.4.2. Pseudotyped virus production and neutralization assays

Two sets of pseudotyped virus were generated using a three-plasmid, second-generation lentiviral system as previously described [93,94]. The first set carried filovirus glycoproteins (GP) from, respectively, Ebola virus (EBOV) Mayinga (EU224440), EBOV Makona (KJ660346.2), Sudan virus (SUDV; FJ968794), Lloviu cuevavirus (LLOV; JF828358), Marburg virus (MARV) Kenya (Z12132) and MARV DRC (DQ447650). A second set carried the fusion (F) and attachment (G) glycoproteins from, respectively, Nipah virus Malaysia (NIVm; AF212302), Nipah virus Bangladesh (NIVb; JN808864.1) and the Ghana virus M74a (HQ660129). Henipavirus and filovirus pseudotyped virus neutralizing titres were determined using endpoint 50% tissue culture infectious dose (TCID_50_) assays [95] on human embryonic kidney 293T clone-17 (HEK 293T/17; American Type Culture Collection (ATCC), CRL-11268) and Chinese hamster ovary K1 (CHO-K1; ATCC, CCL-61), respectively. HEK293T/17 were cultured in a humidified, 37°C, 5% CO_2_ incubator with high glucose Dulbecco's modified Eagle medium (DMEM; Merck) supplemented with 10% fetal bovine serum (FBS; Merck). CHO-K1 cells were cultured in the same conditions using Ham's F-12 nutrient mix (Merck) plus 10% FBS.

A serial dilution (3-fold dilutions; 1 : 50–1 : 12 150) of the bat serum samples was set up in a white 96-well plate (Thermo Fisher), before 100 × TCID_50_ of the specific PV was added. These were incubated for 1 h at 37°C before 2 × 10^4^ of the necessary cells (HEK293T/17 for NIVb and M74a; CHO-K1 for EBOV, SUDV, LLOV and MARV) were added and the plates returned to the incubator for a further 48 h. After this the supernatant was removed and a 50 : 50 mix of Bright-Glo (Promega) and serum-free media added to each well. The luciferase activity was measured in relative light units and neutralizing activity determined using the virus samples that were incubated with a non-specific serum that was negative for antibodies targeting our viral entry protein (VEP) of interest.

### 5.5. RNA sequencing in *Eidolon helvum*

#### 5.5.1. Sample selection

We selected whole blood samples from a subset of *Ei. helvum* individuals (Ebola vaccine group *n* = 8, Nipah vaccine group *n* = 3, PBS group *n* = 2; supplementary material, table S1) for transcriptome analysis. We excluded *Ep. gambianus* because of the low number of bats. For logistical reasons, the samples were selected, processed for RNA extraction and sent for sequencing before the serology results were known. One bat from the PBS-injected group was then revealed to have high antibody response against the Nipah G protein in the MIA before the start of the experiment (figure 2 and supplementary material, figure S5) and was, therefore, excluded from the differential expression analysis, thus leaving one individual as a negative control for the differential expression analysis. As the Nipah vaccine group received an empty vaccine vector as a booster (in contrast to the Ebola vaccine group), gene expression analysis was conducted only up to four weeks post prime vaccine exposure to enable meaningful comparisons between the groups.

#### 5.5.2. RNA extraction and sequencing

RNA was isolated from 150 µl whole blood using the RNAzol® BD kit (Molecular Research Centre, Inc.), following the large RNA isolation protocol with volumes of reagents reduced as follows to account for the small input volume: 300 µl RNAzol® BD, 4.05 µl acetic acid, 450 µl lysate, 22.5 µl 1-bromo-3-chloropropane (BCP), 78.75 µl isopropanol, 60–120 µl 75% ethanol. In step 4, 225 µl supernatant was used. RNA was quantified using a NanoDrop 2000 (Thermo Fisher), and RNA integrity was determined using a 2100 Bioanalyzer (Agilent Technologies, USA). Samples were paired-end-sequenced by Novogene, UK with 150 bp read length on an Illumina sequencer (Illumina, USA). The average number of raw sequencing reads per sample was 26.25 ± 3.02 Mb (mean ± s.d. PE150).

#### 5.5.3. Quality check and read mapping

The quality of the RNA sequencing data was evaluated using the FastQC software v. 0.11.4 [96], and low-quality sequences were trimmed using the bbduk.sh script from the BBTools bioinformatics package (https://sourceforge.net/projects/bbmap/). First, over-represented sequences as detected during the quality check and adaptor sequences (ref = adapters.fa.gz) were removed. Approximately 75% of the transcripts from the blood samples mapped to haemoglobin and were thus removed from the dataset. Further, the leftmost 10 bases of the sequencing reads (ftl = 10), and both ends (qtrim = rl) were trimmed until the average read quality (Phred score) was 10 or greater (trimq = 10). Finally, any reads shorter than 25 bp (minlen = 25) were discarded. After removal of over-represented sequences and trimming, an average of 6.82 ± 3.08 Mb sequencing reads remained for the blood samples. The quality filtered reads from each blood sample were then aligned to an in-house-generated *Ei. helvum* draft genome assembly and annotation file (available on figshare [57]) and the gene expression level quantified using STAR v. 2.7.3a with default options and –quantMode GeneCounts [97]. The percentage of trimmed sequencing reads that mapped to a unique location in the *Ei. helvum* genome ranged from 76 to 88%.

Owing to low-quality RNA from the PBS-injected bat taken at two weeks post injection, no comparison was made between the PBS- and vaccine-injected (treatment) groups at two weeks post prime injection. The differential expression analysis was thus conducted using data from one PBS-injected individual, two or three Nipah vaccine-treated bats and six to eight Ebola vaccine-treated bats depending on the time point (figure 5A and supplementary material, table S1).

#### 5.5.4. Principal component analysis

A principal component analysis (PCA) was used to visualize sample-to-sample distances solely on the basis of their gene expression profiles using the plotPCA function in DESeq2 v. 1.36.0 [98] in R v. 4.2.1 [99]. One control individual with high anti-Hendra virus antibody levels (supplementary material, figure S5) clustered as an outlier in the PCA (supplementary material, figure S20) and was, therefore, excluded from the differential expression analysis, leaving a single PBS individual for all comparisons. A temporal pattern was observed in the transcriptomic data (supplementary material, figure S20), which was controlled for by comparing PBS-injected and vaccine-treated groups for each time point separately.

#### 5.5.5. Differential gene expression analysis

To investigate which genes were up- or down-regulated in response to each vaccine, gene expression levels between the PBS group and each vaccine-treated group were compared in blood samples at each time point (one to four weeks post prime vaccine exposure). To identify genes that were differentially expressed between the Nipah and the Ebola vaccine group, gene expression levels were further compared between each vaccine group in blood samples from one to four weeks post prime vaccine exposure. All differential gene expression analyses were conducted in DESeq2 v. 1.36.0 [98], which takes the low number of replicates into account [100]. *p*-values were adjusted using the Benjamini and Hochberg false discovery rate (FDR) correction for multiple testing [101]. Genes with an FDR less than 0.05 were considered differentially expressed.

The top DEGs from each time point for each vaccine group were identified, and their fold change visualized in heat maps using the pheatmap package [102]. Upset plots [103] were then used to explore whether the same genes were differentially expressed at different time points. The results from the differential expression analysis between the Nipah and the Ebola vaccine group were visualized in volcano plots using the R package EnhancedVolcano v. 1.14.0 [104]. To investigate whether the DEGs from this comparison were up- or down-regulated in each vaccine group when compared with the PBS group, the log2 fold change for each vaccine group when compared with the PBS group was plotted. GO analyses [56] in the WebGestalt 2019 online tool [105] were performed to investigate if any biological processes or KEGG pathways [63] were over-represented in the lists of DEGs. Over-represented GO terms were visualized in heat maps using the pheatmap package [102]. To investigate whether the DEGs from the over-represented GO terms were up- or down-regulated in the vaccine-exposed bats, the log2 fold change of the DEGs from over-represented GO terms was further visualized in heat maps using the pheatmap package [102].

## Supplementary Material



## Data Availability

Raw mRNA-seq data have been deposited on the NCBI's Sequence Read Archive (SRA) database with accession number PRJNA1054324. The in-house-generated draft genome assembly and annotation file are available on Figshare [57] (doi:10.6084/m9.figshare.29117609.v2) and the data used to generate them on NCBI SRA (PRJNA1054324). Datasets S1–S3 in the supplementary material as well as the R code for reproducing the main serological and differential expression analyses have been deposited on Figshare [66] (doi:10.6084/m9.figshare.29126510.v2). Supplementary material is available online [106].
