# Supplementary material for: Serological and transcriptomic response of African fruit bats to immune challenge with recombinant vaccines expressing Ebola or Nipah virus antigens
Source: R Soc Open Sci. 2026 Oct 7;13(10):252189. doi: 10.1098/rsos.252189 (PMC13641923; doi:10.1098/rsos.252189)
Supplement: Supplementary file 1 [file rsos.252189.zip › rsos.252189/rsos252189_si_001.docx]

Figure Captions for submission of manuscript RSOS-252189.R2

Serological and transcriptomic response of African fruit bats to immune challenge with recombinant vaccines expressing Ebola or Nipah virus antigens

Figure 1: Timeline of serum sample collection by species and by experimental group. The two injections were given four weeks apart, with the ChAdOx1 vaccine given at week 0 and the MVA vaccine given at week 4. The attrition in *Epomophorus gambianus* numbers was due to deaths occurring in all three groups. w = weeks after first injection.

Figure 2: Anti-Nipah-G antibody response in all treatment groups A) Time series of mean log10(MFI) values against Nipah-G, by species and experimental group. As no change in anti-Nipah-G antibodies was expected in the Ebola vaccine group, this group is considered a control in this figure. Each line represents one individual bat. Vertical dotted lines show the dates of injections in each group. Samples and timepoints for which transcriptomic data was included in the differential gene expression analysis are shown in dark orange. B) Boxplot showing the log change in Nipah-G MFI values between the initial and third week (pre-booster) for each *Eidolon helvum* treatment group. Points represent the net change across that period for each *E. helvum* bat. C) Boxplot showing the log change in Nipah-G MFI values between the initial and eighth (final) week for each *E. helvum* treatment group. Points represent the net change across that period for each *E. helvum* bat.

Figure 3. Serological response dynamics and correlations in *Eidolon helvum* bats following Nipah-G vaccine delivery. A) Association between the initial Nipah-G log-MFI value and the change in log-MFI values across the entire study period. Each dot represents a single *Eidolon helvum* bat. B) Boxplot showing the changes in log-MFI values for all tested antigens across the entire study period for *E. helvum*. C) Correlation between the MFI values from the Luminex-based multiplex microsphere immunoassay (MFI values against the Nipah-G antigen) and IC50 values from the lentivirus pseudotyped virus neutralisation assay (serum dilution required to achieve 50% neutralization). The starting dilution was 50-fold; four sera with an IC50 below 50-fold are shown as points with a value of 50 (~1.7 on the log scale).

Figure 4: Anti-Ebola-GP1 antibody response in all treatment groups Time series of mean log10(MFI) values against Ebola, by species and experimental group. As no change in anti-Ebola-GP1 antibodies was expected in the Nipah vaccine group, this group is considered a control in this figure. Vertical dotted lines show the dates of injections in each group. Samples and timepoints for which transcriptomic data was included in the differential gene expression analysis are shown in dark orange. B) Boxplot showing the log change in Ebola-GP1 MFI values between the initial and third week (pre-booster) for each *Eidolon helvum* treatment group. Points represent the net change across that period for each *E. helvum* bat. C) Boxplot showing the log change in Ebola-GP1 MFI values between the initial and eighth (final) week for each *E. helvum* treatment group. Points represent the net change across that period for each *E. helvum* bat.

Figure 5: Differentially expressed genes in the *Eidolon helvum* vaccine recipients. A) Number of differentially expressed genes for the Ebola and Nipah vaccine treatment groups for each timepoint (FDR < 0.05) when compared to the PBS individual. 1w-4w: blood samples collected weekly. Sample size for each vaccine group is displayed above the barplot. The PBS group had one sample per time point. B) UpSet plot of the intersection of differentially expressed genes (DEGs) in the Ebola and Nipah vaccine treatment groups. The nature of a given intersection is indicated by the dots below the bar plot. For instance, the genes (n=968) in the first column were differentially expressed in the bats retrieving the Ebola vaccine but not the Nipah vaccine. C-D) Heatmap illustrating the log2 fold change of the top ten differentially expressed genes (rows) from each timepoint in the (C) Ebola and (D) Nipah vaccine groups. Red shading indicates that gene expression was higher-, and blue shading indicates that gene expression was lower in the vaccine group than in the PBS group. Grey colour indicates time points when the genes were not significantly differentially expressed. Genes that could not be assigned a gene name from the in-house generated *Eidolon helvum* draft genome are labelled as unknown. All DEGs from the vaccine groups are listed in electronic supplementary datasets S1-2 [62].

Figure 6: log_2_ fold changes of the expression level between the *Eidolon helvum* Ebola vaccine and PBS group mapped on the Th1 and Th2 cell differentiation pathway (GO:hsa04658) from the KEGG database [64-66]. Red shading indicates that gene expression was significantly higher, and blue shading indicates that gene expression was significantly lower in the Ebola vaccine group than in the PBS group (*n_PBS_*=1, *n*_ebola_=6-8, for exact number of individuals see figure 5A). Genes not significantly differentially expressed are shown in grey for that particular time point. Each box represents one gene in the pathway and the columns within the box show the gene expression fold change for blood samples at the different time points; 1w, 3w, and 4w post prime vaccine, from left to right. Data is shown for genes that were differentially expressed at least one time point. w = weeks

Figure 7: A) Volcano plot displaying log2 fold change in gene expression between the *Eidolon helvum* Nipah and Ebola vaccine groups versus log10 transformed p-value one week after prime vaccine exposure. Genes from over-represented KEGG pathways [64] (electronic supplementary files, figure S12) are visualised in colour (n_ebola_=6-8, n_nipah_=2-3, for exact number of individuals see figure 5A). B) log2 fold change of genes from each vaccine group compared to the PBS group. Orange dots denote significantly differentially expressed genes between the Nipah and Ebola vaccine groups, dark orange dots denote significantly differentially expressed genes with a log2 fold change of at least 2, and triangles denote differentially expressed genes up-regulated in one vaccine group and down-regulated in the other when compared to the PBS group (*n*_ebola_=6-8, *n*_nipah_=2-3, *n*_control_=1, for exact number of individuals see figure 5A). Data from other timepoints are shown in the electronic supplementary files (figures S15-17)

Figure 8: Differential gene expression between the *Eidolon helvum* Ebola and Nipah vaccine groups one week after initial vaccine exposure mapped to the ISGylation pathway. log2 fold changes in gene expression are displayed for genes involved in the ISGylation signaling pathway (*n*_ebola_=6-8, *n*_nipah_=2-3, for exact number of individuals see figure 5A). As described in the literature, ISG15 is attached to substrates in a three-step enzymatic (E) cascade. First it is activated by E1 UBA7, then it is transferred to the E2 UBE2L6 and subsequently to a target protein, such as a viral protein, through the E3 HERC5 [67]. Purple shading indicates significantly lower gene expression in the Ebola vaccine group than in the Nipah vaccine group, while genes not significantly differentially expressed (*UBE2L6*) are shown in grey. *UBA7* has three different shadings as reads mapped to three different locations on our in-house generated *Eidolon helvum* genome assembly, all annotated as UBA7. UBA7a = EHELV G00034959, UBA7b = EHELV_G00003507, UBA7c = EHELV_G00003508
