# Supplementary material for: Serological and transcriptomic response of African fruit bats to immune challenge with recombinant vaccines expressing Ebola or Nipah virus antigens
Source: R Soc Open Sci. 2026 Oct 7;13(10):252189. doi: 10.1098/rsos.252189 (PMC13641923; doi:10.1098/rsos.252189)
Supplement: Supplementary file 1 [file rsos.252189.zip › rsos.252189/rsos252189_si_002.pdf]

Supplementary files for

Serological and transcriptomic response of African fruit bats to immune challenge with recombinant vaccines expressing Ebola or Nipah virus antigens

Contents:

Section 1. Supplementary files (serology and gene expression)

- Supplementary texts S1-5

- Supplementary figures S1-20

- Supplementary tables S1-2

Section 2. Supplementary files (viral vectors)

Text S1. Mixed-effect model

As an alternative method to detect changes in antibody levels over the course of the experiment, we fitted linear mixed-effects models with log-MFI as the response variable, vaccine group and day as fixed effects, and bat identity as a random effect to account for repeated measurements. We tested log-MFI for Nipah and Ebola antigens separately, in each case comparing the bats receiving the matching vaccine to the rest.

<u>Model one, Nipah vaccine</u>

```
lme(Nipah_G_meanlog ~ Group*Day, eidolon.Nipah_G.meanlog, random= ~Day|Bat_ID)
```

	**Value**	**Std.Error**	**DF**	**t-value**	**p-value**
(Intercept)	2.1755581	0.2225542	188	9.775406	0.0000
GroupNipah_G_vaccine	-0.3938323	0.3666291	17	-1.074198	0.2977
Day	-0.0036630	0.0024220	188	-1.512361	0.1321
GroupNipah_G_vaccine:Day	0.0232642	0.0039885	188	5.832866	0.0000

<u>Model two, Ebola vaccine</u>

```
lme(Ebola_meanlog ~ Group*Day, eidolon.ebola.meanlog, random= ~Day|Bat_ID)
```

	**Value**	**Std.Error**	**DF**	**t-value**	**p-value**
(Intercept)	1.4560733	0.08896233	188	16.367303	0.0000
GroupEbola_vaccine	0.0247616	0.13715029	17	0.180543	0.8589
Day	-0.0025147	0.00123446	188	-2.037063	0.0430
GroupEbola_vaccine:Day	0.0018846	0.00190479	188	0.989380	0.3237

Text S2. Pseudotype neutralisation assay

Pseudotype virus (PV) production

PVs were generated using a three plasmid, second generation lentiviral system as previously described [PMID: 19925950 and PMID: 34449756]. Viral envelope proteins (VEP) were cloned into pCAGGS [PMID: 1660837] meaning their expression was driven by a chicken beta-actin promoter. PVs were generated bearing glycoproteins (GP) from Ebola virus (EBOV) Mayinga (EU224440), EBOV Makona (KJ660346.2), Sudan virus (SUDV; FJ968794), Lloviu cuevavirus (LLOV; JF828358), Marburg virus (MARV) Kenya (Z12132) and MARV DRC (DQ447650). The F&G proteins from Nipah virus Malaysia (NIVm; AF212302), Nipah virus Bangladesh (NIVb; JN808864.1) and the Ghanian bat paramyxovirus M74a (HQ660129) were also used to generate PVs. Paramyxovirus and filovirus PV titres were determined using endpoint TCID50 assays[1] using human embryonic kidney 293T clone-17 (HEK 293T/17; American type culture collection (ATCC), CRL-11268) and Chinese hamster ovary K1 (CHO-K1; ATCC, CCL-61) cells respectively. HEK293T/17 were cultured in a humidified, 37°C, 5% CO2 incubator with high glucose Dulbecco's Modified Eagle Medium (DMEM; Merck) supplemented with 10% foetal bovine serum (FBS; Merck). CHO-K1 were cultured in the same conditions using Ham's F-12 nutrient mix (Merck) plus 10% FBS.

PV neutralisation assays (PVNA)

A serial dilution (3-fold dilutions; 1:50 - 1:12,150) of the bat serum samples was set up in a white 96- well plate (ThermoFisher), before 100 x TCID50 of the specific PV was added. These were incubated for 1h at 37°C before 2x104 of the necessary cells (HEK293T/17 for NIVb and M74a; CHO-K1 for EBOV, SUDV, LLOV and MARV) were added and the plates returned to the incubator for a further 48h. After this the supernatant was removed and a 50:50 mix of BrightGlo (Promega) and serum-free media added to each well. The luciferase activity was measured in relative light units and neutralising activity determined using the virus samples that were incubated with a non-specific serum that was negative for antibodies targeting our VEP of interest.

1 R. C. Condit. Principles of virology. In David M Knipe and Peter M Howley, editors, Fields virology. Lippincott Williams & Wilkins Philadelphia, Philadelphia, 2001.

Text S3. In vitro cell entry assay

MVA and ChAdOx1 infection assays

To assess the ability of the vaccine vectors (ChAdOx1 and MVA) to infect bats and express the antigens, we used established bat cell lines and modified ChAdOx1 and MVA vectors carrying a green fluorescence protein (GFP) reporter gene. For this purpose, stocks of MVA-GFP and ChAdOx1-GFP were titrated onto known permissive cell lines (HEK293T/17 and A549 [ATCC CCL-185]) as well as *E. helvum* lung (HELV-L; provided by Ash Banyard, APHA) and kidney (HELV-K; also provided by Ash Banyard, APHA), *E. gambianus* (EG81; provided by Michelle Baker, CSIRO) and *R. aegyptiacus* (R06E; BEI NR-49168) cell lines. The A549 cells were maintained in DMEM plus 10% FBS, HELV-L and -K in DMEM plus 10% FBS and 100U/ml penicillin, 100 µg/ml streptomycin (pen/strep), and the EG81 and R06E in DMEM/F12 Ham's Media (Merck) with 10% FBS, pen/strep and 2.5 µg/ml amphotericin B. Following inoculation, cells were incubated for 48h before the level of infection was assessed via flow cytometry detection of GFP positive cells. These *in vitro* infection assays showed that both ChAdOx1 and MVA vectors were able to infect *E. helvum* and *E. gambianus* (as well as *Rousettus aegyptiacus*) cells with equal efficiency (electronic supplementary files, figure S4).

Text S4. Differentially expressed genes from the T-helper (Th) 1 and Th2 cell differentiation pathway

The majority of the differentially expressed genes from the Notch signalling pathway were down-regulated in the Ebola vaccine-exposed bats (electronic supplementary files, figure S12), including the notch receptors *Notch1* and *Notch2*, several notch signalling modifiers (Fringe and Dishevelled genes), and several genes from the co-repressor complex (i.e., two homologues of the notch pathway corepressor *Nuclear Receptor Corepressor 2*, *NCOR2*). In contrast, *HDAC2* (*Histone Deacetylase 2*) and *SNW1* (*SNW Domain C E. helvum ontaining 1*) which both regulate the expression of Notch target genes were upregulated in the blood of Ebola vaccine exposed bats. Several members of the JAK and STAT families involved in the Th1 and Th2 cell differentiation pathway (*JAK2*, *STAT1* and *STAT4*) were up-regulated in the Ebola vaccine-exposed bats (Figure 6), as was the gene encoding one of the subunits of the *interleukin 12* (*IL-12*) receptor (*IL-12R beta 1*). The IL-12 receptor activates the Janus kinases (Tyk2 and JAK2) and the transcription factor STAT4, which up-regulates IFN-gamma and IL-12RB2 gene expression.

Text S5. Additional differentially expressed genes and overrepresented GO-terms

Several genes that encode proteins involved in regulation of apoptosis (i.e., *AATK* and *RERE*[2,3]) were also among the top differentially expressed genes between the Ebola and Nipah vaccine groups (electronic supplementary files, figure S18). These genes had lower expression level in the Ebola vaccine group than in the Nipah vaccine group, and were significantly down regulated in the Ebola vaccine group when compared to the control group (electronic supplementary files, figure S18, electronic supplementary datasets S2-3).

Several GO terms related to type I interferon response, response to virus, T cell selection, Notch signaling and JAK/STAT signaling were over-represented in the genes that were differentially expressed between the Nipah and Ebola vaccine groups (electronic supplementary files, figure S13). In most cases, differentially expressed genes from over-represented immune related GO terms had lower expression levels in the Ebola vaccine group than in the Nipah vaccine group (i.e., the interferon stimulated genes Interferon-induced GTP- binding protein *Mx1* and *Radical S-adenosyl methionine domain-containing protein 2*, electronic supplementary files, figure S14). Some exceptions were genes encoding the *signaling lymphocytic activation molecule 1* (*SLAMF1*), which has been implicated in the response to Ebola virus vaccines previously[4], *exportin 1* (*XPO1*), which is important for nuclear export/import in henipavirus infection[5], and *Influenza virus NS1A-binding protein* (*IVNS1ABP*) (electronic supplementary files, figure S14).

2 Eugenia Gaozza, Stacey J Baker, Renu K Vora, and E Premkumar Reddy. Aatyk: a novel tyrosine kinase induced during growth arrest and apoptosis of myeloid cells. Oncogene, 15(25):3127–3135, 1997.

3 Thomas Waerner, Paola Gardellin, Klaus Pfizenmaier, Andreas Weith, and Norbert Kraut. Human rere is localized to nuclear promyelocytic leukemia oncogenic domains and enhances apoptosis. Cell Growth Differ, 12(4):201–210, 2001.

4 Andrea R Menicucci, Allen Jankeel, Heinz Feldmann, Andrea Marzi, and Ilhem Messaoudi. Antiviral innate responses induced by vsv-ebov vaccination contribute to rapid protection. MBio, 10(3):10–1128, 2019.

5 Celine Deffrasnes, Glenn A Marsh, Chwan Hong Foo, Christina L Rootes, Cathryn M Gould, Julian Grusovin, Paul Monaghan, Michael K Lo, S Mark Tompkins, Timothy E Adams, et al. Genome-wide sirna screening at biosafety level 4 reveals a crucial role for fibrillarin in henipavirus infection. PLoS Pathogens, 12(3):e1005478, 2016.

Figure S1. Serial dilutions of virus-specific monoclonal antibodies as positive controls in the Luminex MIA. Four monoclonal antibodies, matching the viruses being tested by the Luminex MIA, were used as positive controls in serial dilutions. 5D2 against Ebola virus glycoprotein (*Orthoebolavirus zairense*), 2B8 (murine) against Marburg virus (*Orthomarburgvirus marburgense*), 102.4 against Hendra/Nipah G protein (*Henipavirus hendraense* and *Henipavirus nipahense*), 14F3 against Cedar virus (*Henipavirus cedarense*)

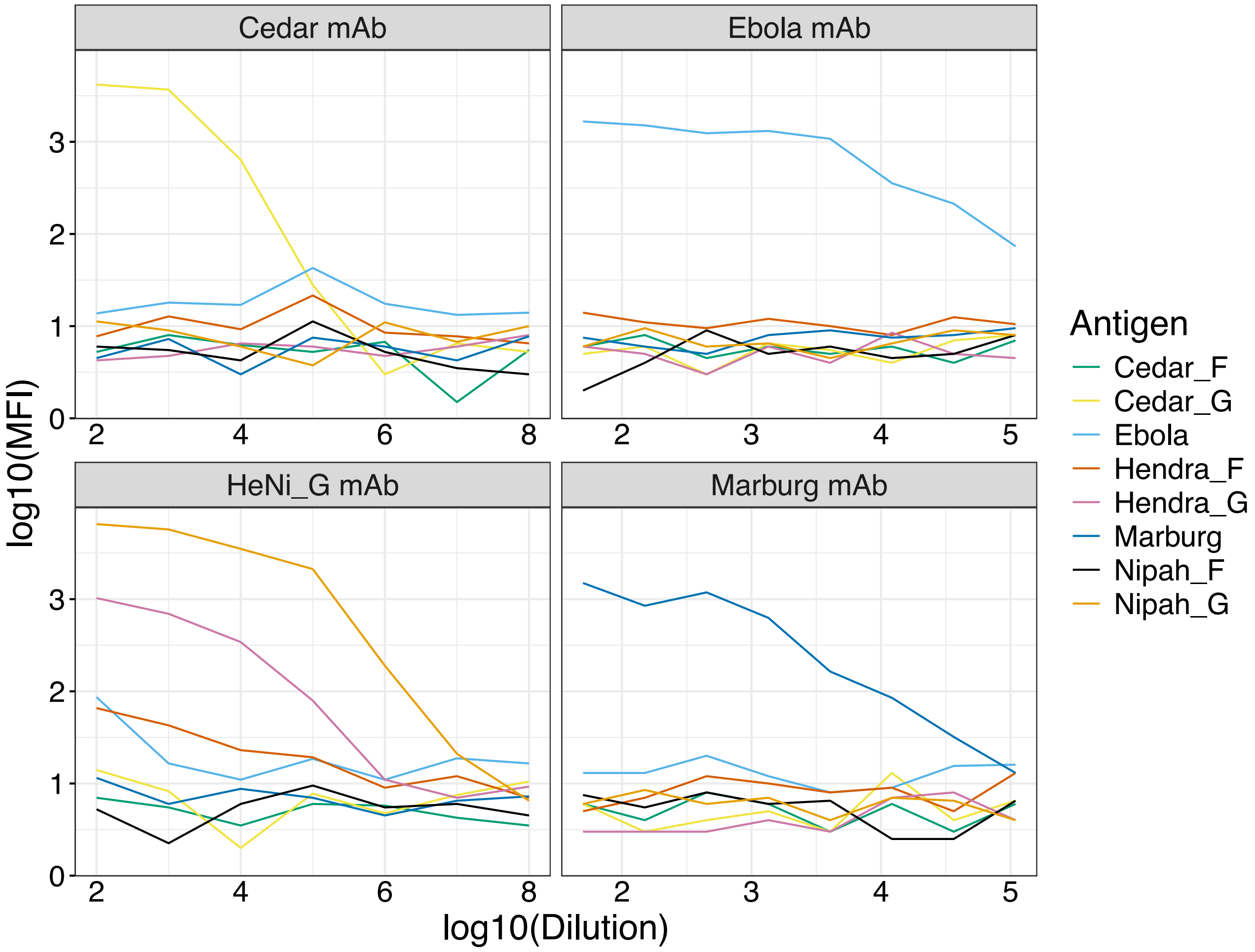

Figure S2. Time series of mean log10(MFI) values across antigens in *Eidolon helvum* by experimental group. Each coloured line represents an individual bat. Within each treatment group, measurements across antigens are derived from the same individuals; however, no individuals are shared between treatment groups, so each column represents a different set of individuals. Vertical bars at each sampling time point indicate the variation between two technical replicates measured from the same biological sample in the Luminex xMAP-based multiplex microsphere-based immunoassay. Vertical dotted lines mark the timing of injections for each experimental group.

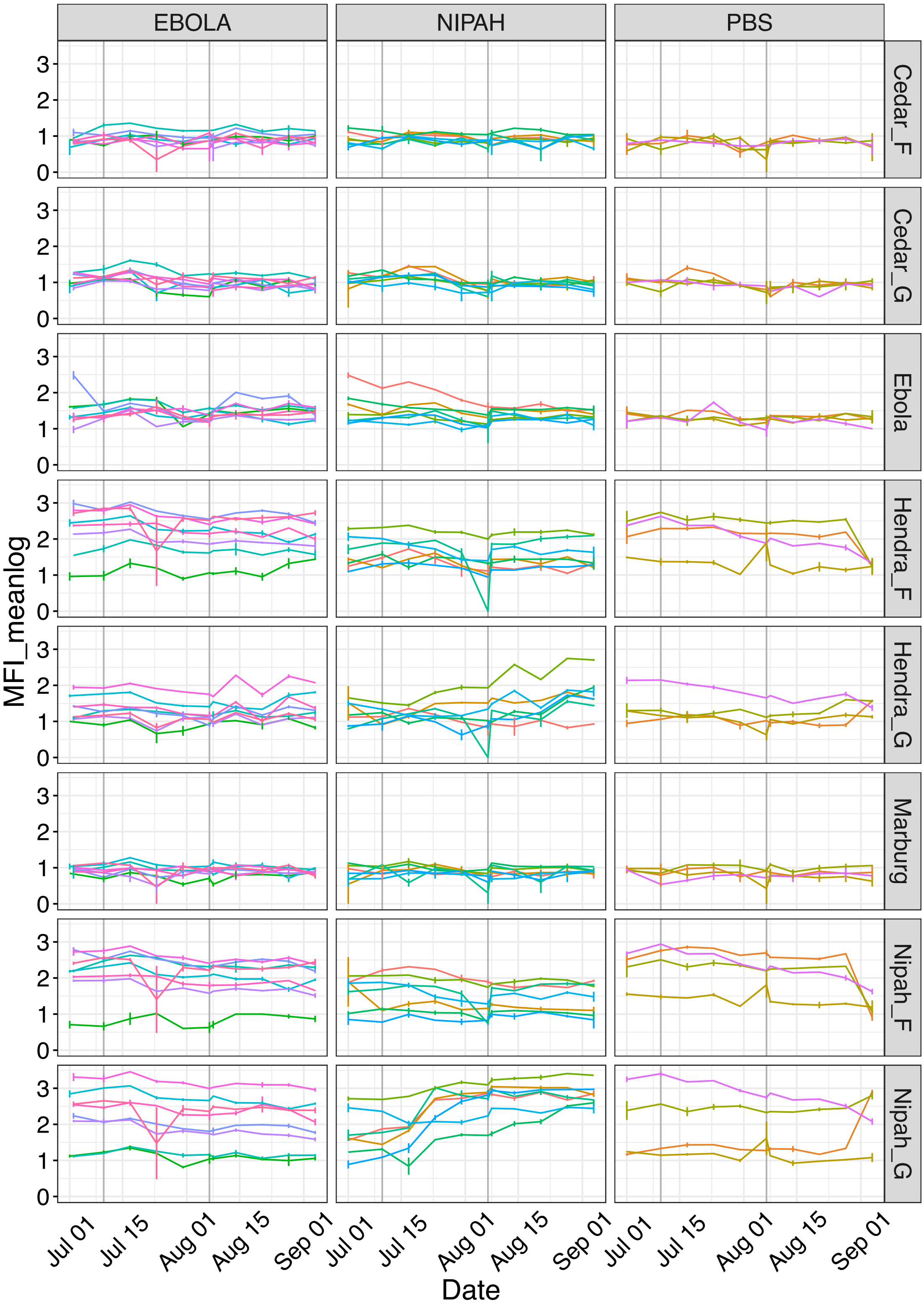

Figure S3. The meanlog MFI values measured with the Luminex xMAP-based multiplex microsphere-based immunoassay for each antigen at the time of capture.

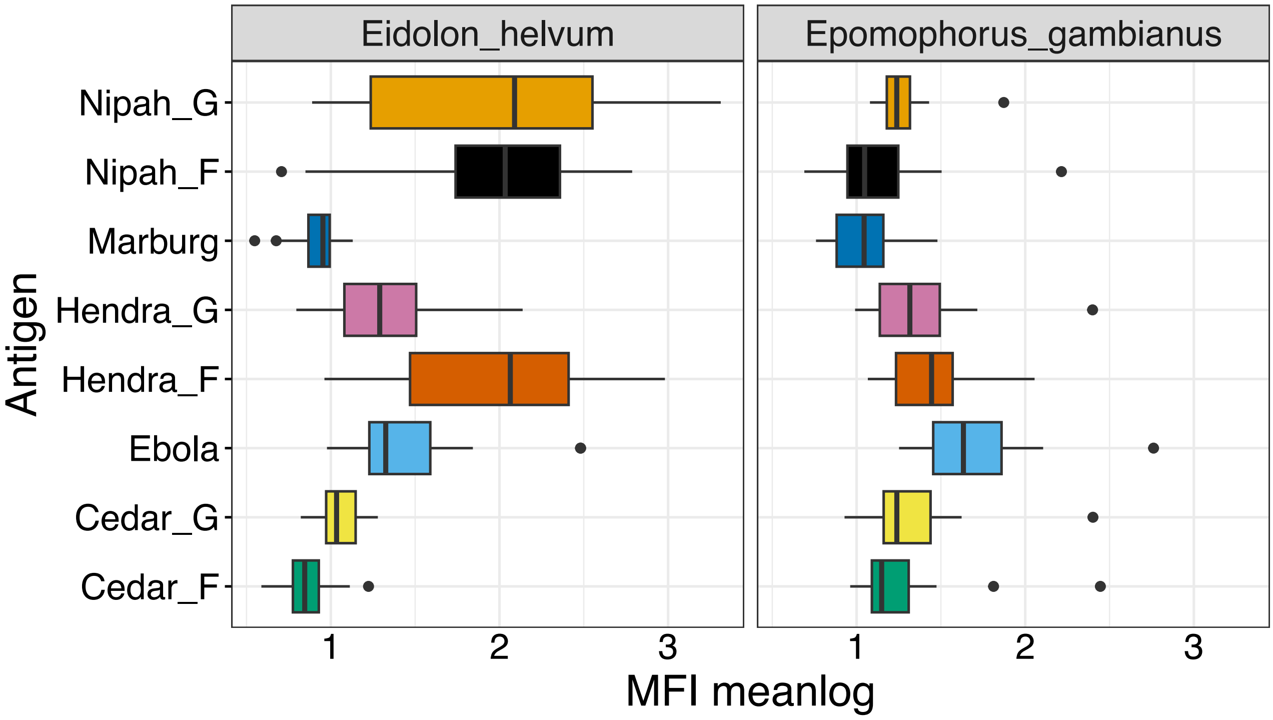

Figure S4. Infection assays showed that MVA and ChAdOx were able to infect *E. helvum* and *E. gambianus* (and *R. aegyptiacus*) cells. Grey bars show known permissive human cell lines. Error bars are the standard error of the mean.

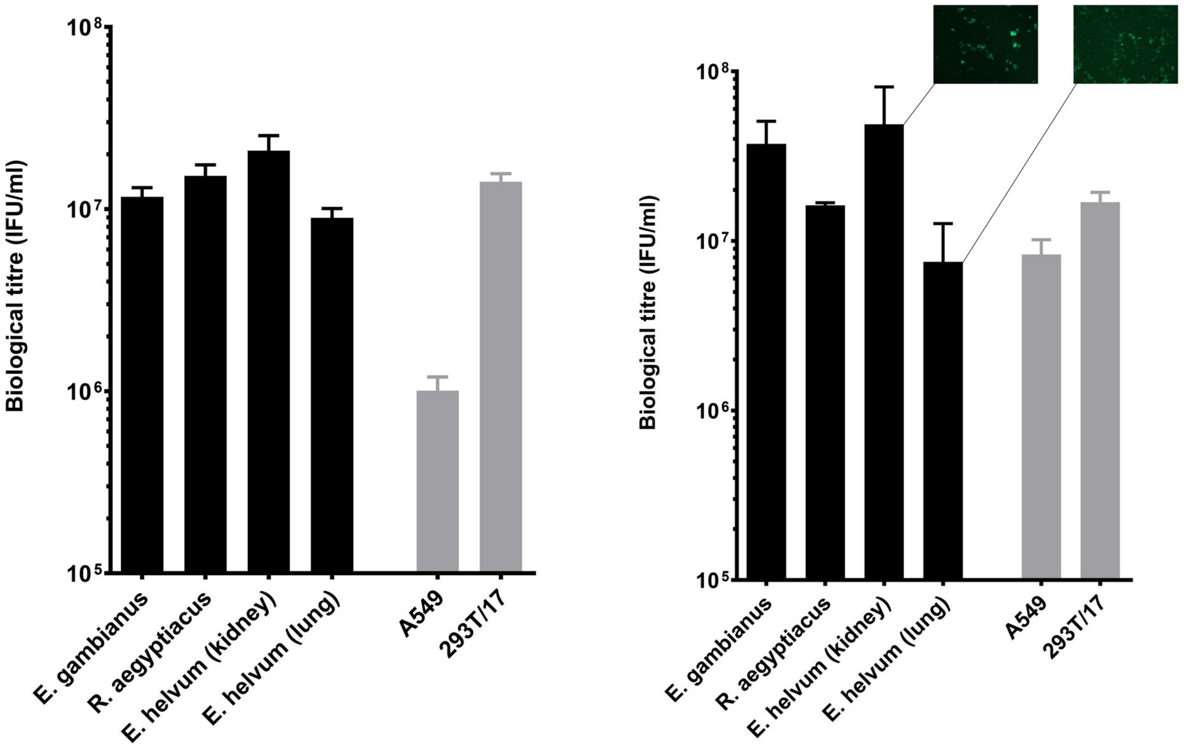

Figure S5: Level of antibodies against Ebolavirus and henipaviruses before and after vaccination in individuals for which gene expression was measured. The antibody levels displayed are median fluorescence intensities (MFI) measured using a Luminex multiplexed binding assay. 1w-8w: blood samples collected weekly. The control individual with high affinity against the Nipah G protein before the start of the experiment was excluded from the differential expression analysis thus leaving one single individual in the control group. Gene expression was assessed only until week four, which was at least three weeks before the second individual's antibody levels increased.

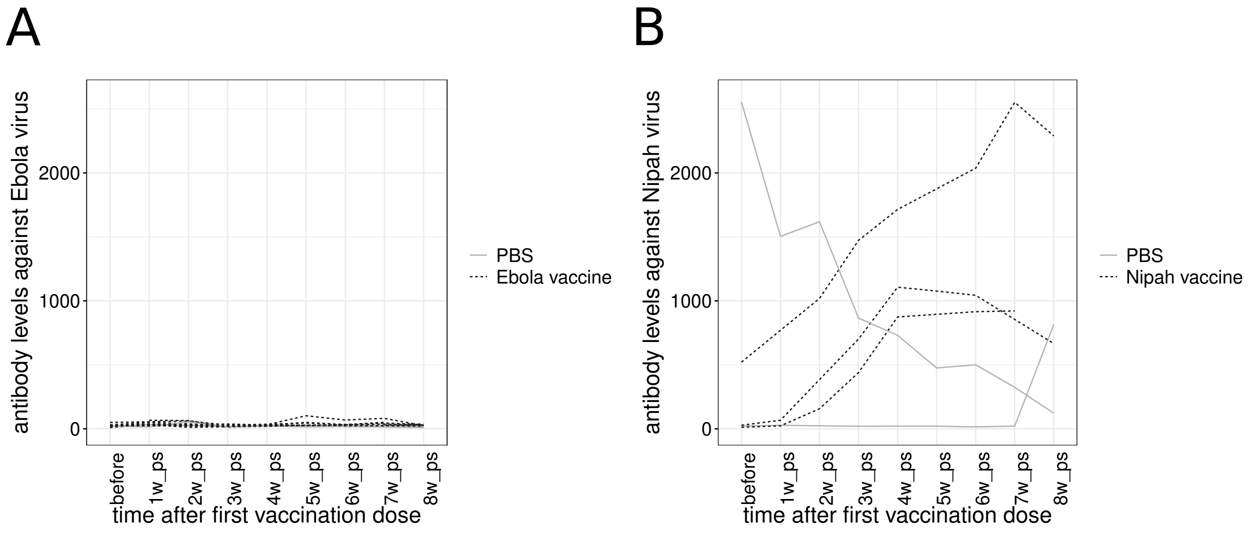

Figure S6. Heatmap illustrating the log2 fold change in expression of genes (rows) that were significantly differentially expressed at least once in both the Ebola and Nipah vaccine groups following prime vaccine exposure but prior to booster administeration. Red shading indicates that gene expression was higher-, and blue shading indicates that gene expression was lower in the vaccine group than in the PBS group. Grey colour indicates time points when the genes were not significantly differentially expressed.

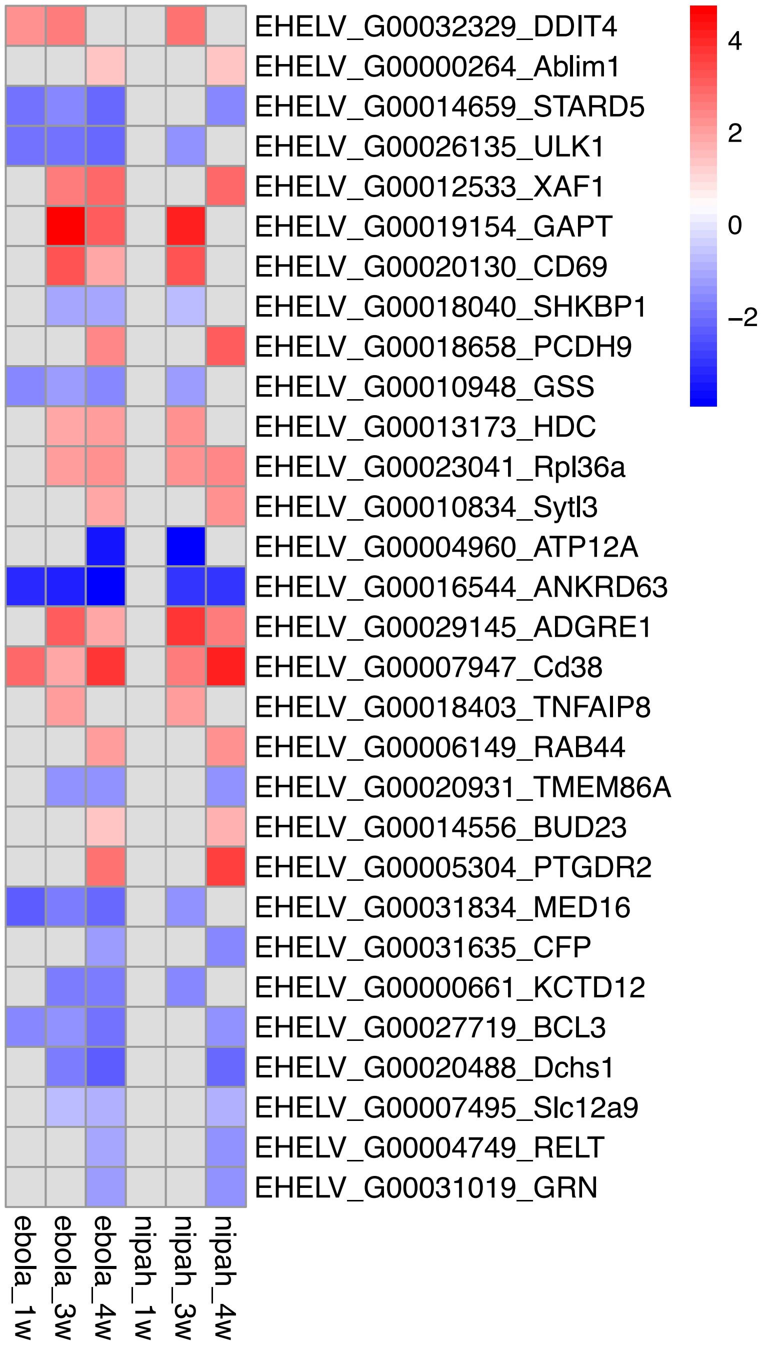

Figure S7. Heatmap illustrating the log2 fold change in expression of genes (rows) that were uniquely significantly differentially expressed at least once in the Nipah vaccine groups following prime vaccine exposure but prior to booster administeration. Red shading indicates that gene expression was higher-, and blue shading indicates that gene expression was lower in the vaccine group than in the PBS group. Grey colour indicates time points when the genes were not significantly differentially expressed

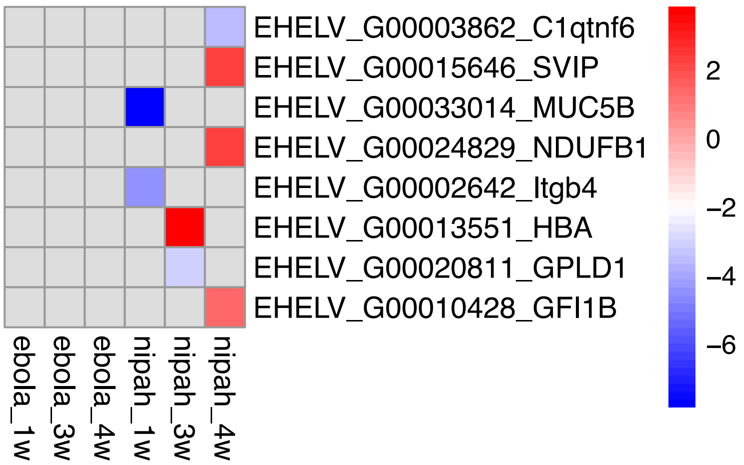

Figure S8: Overlap of differentially expressed genes for the Ebola and nipah vaccine treatment groups for each timepoint. 1w-4w: blood samples collected weekly.

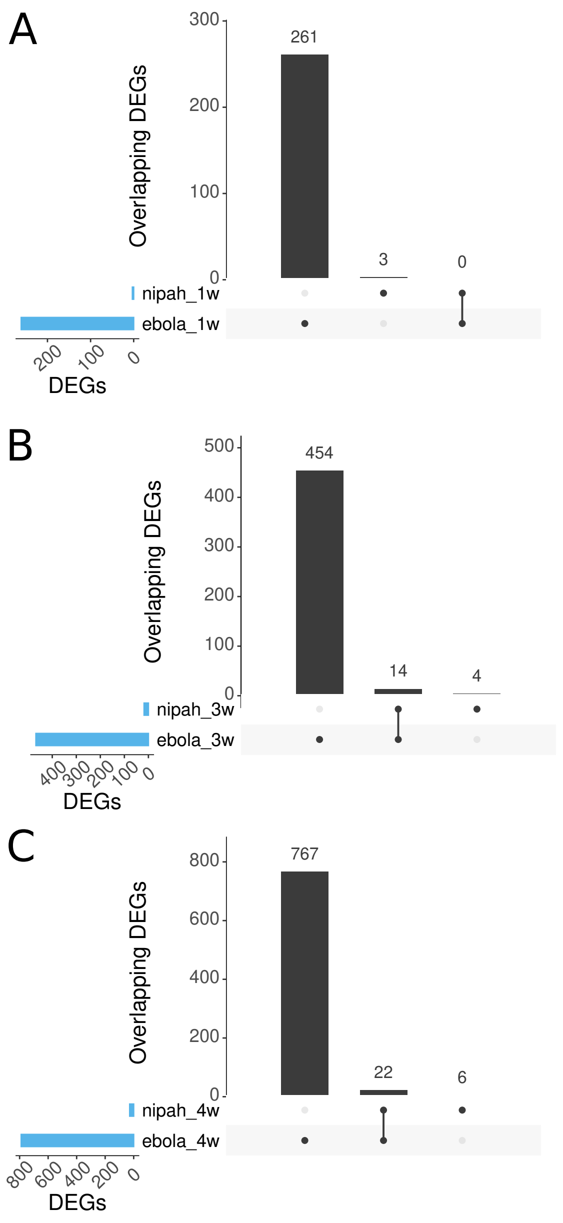

Figure S9: Overlap of differentially expressed genes in blood over the course of the experiment for each vaccination group. 1w-4w: blood samples collected weekly.

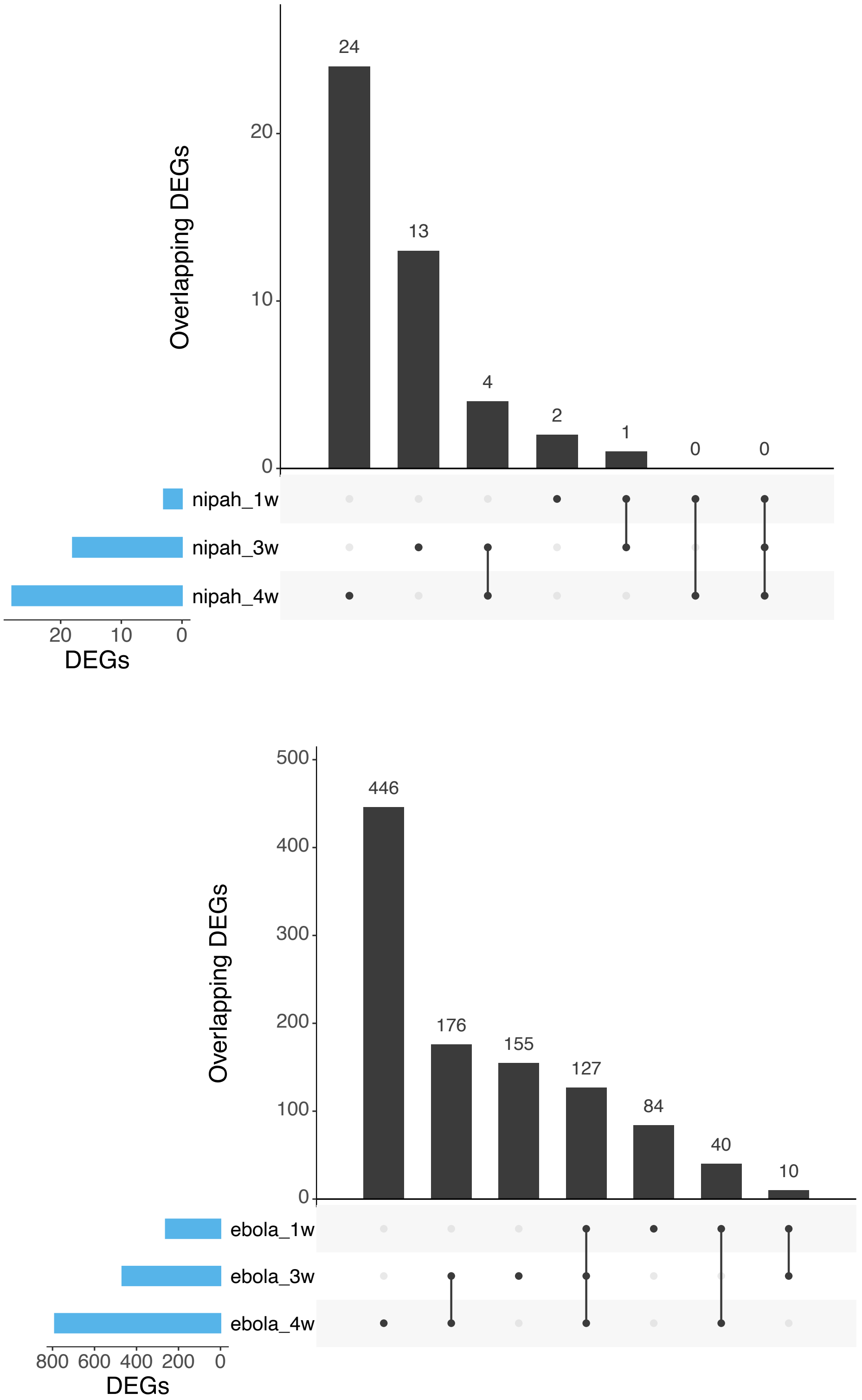

Figure S10: Overrepresented KEGG pathways in the gene expression data from the vaccinated bats. Heatmaps illustrating overrepresented pathways for the Ebola and nipah vaccine treatment groups. Pathways that were significantly overrepresented (FDR<0.05) are shown in colour, with darker red indicating a higher enrichment score. Pathways that were not significantly overrepresented or differentially expressed are shown in grey for that particular treatment group and time point.

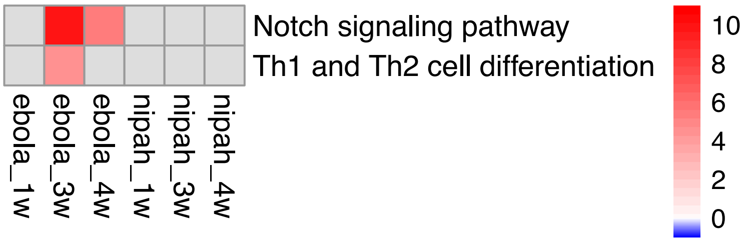

Figure S11: Overrepresented biological processes (BPs) in the gene expression data from the Ebola vaccination group. Biological processes (BPs) that were significantly overrepresented (FDR<0.05) are shown in red colour, with faint red indicating lower fold enrichment score and dark red a higher fold enrichment score. Pathways that were not significantly overrepresented are shown in grey for that particular treatment group and time point. 1w-4w: blood samples collected weekly

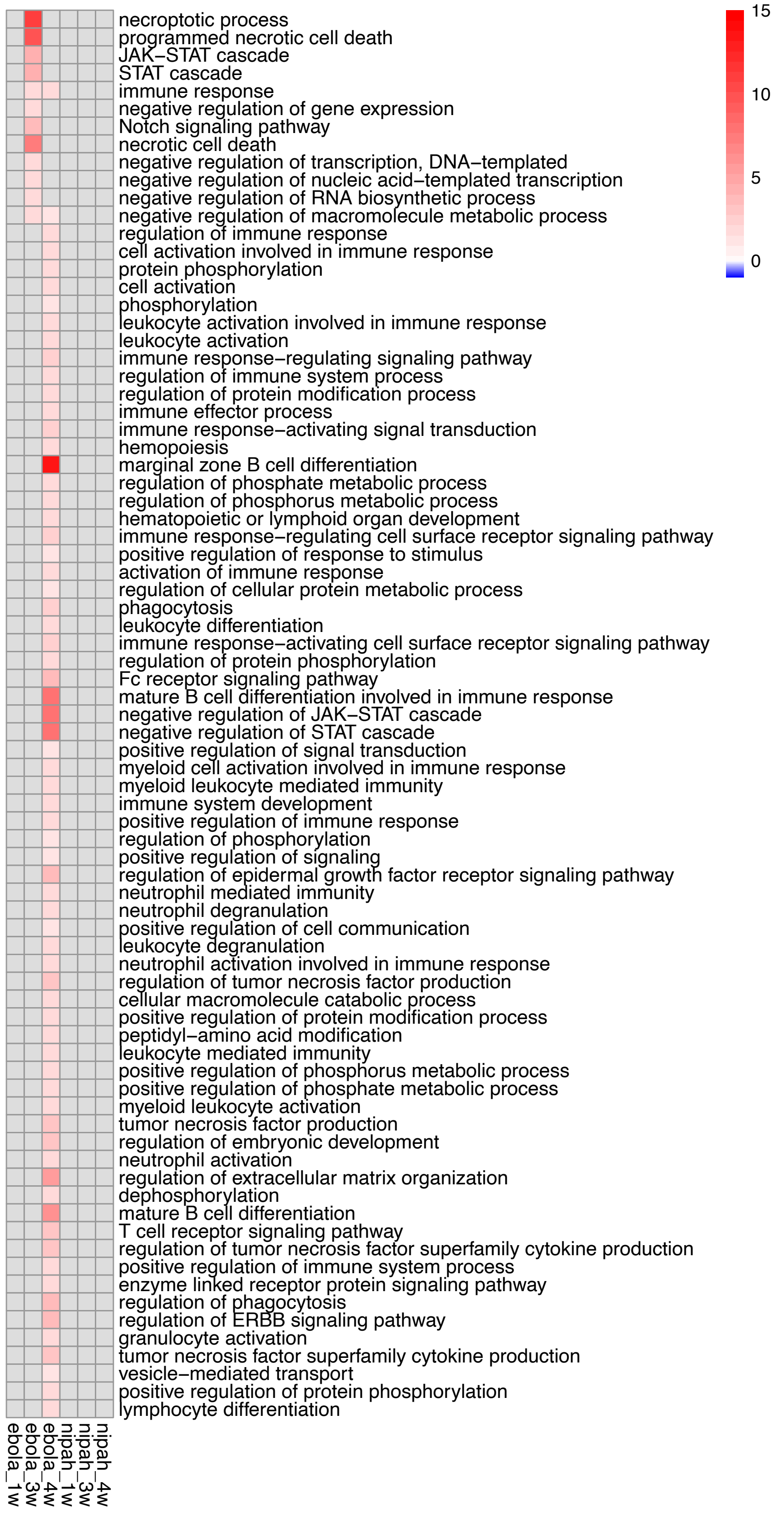

Figure S12: Log2 fold changes of the expression level between the Ebola vaccine and control group mapped on the Notch signaling pathway (GO:hsa04330) from the KEGG database[6]. Red indicates that the gene expression was significantly higher, and blue indicates that the gene expression was significantly lower in the Ebola vaccine treatment group than in the control group. Genes that were not significantly differentially expressed are shown in grey for that particular time point. Each box represents one gene in the pathway and the columns within the box show the gene expression fold change for blood samples at the different time points; 1w, 3w and 4w ps from left to right. Data is shown on genes that were differentially expressed at at least one time point. NCOR2a = EHELV G00014202, NCOR2b = EHELV G00014201, NOTCH2a = EHELV G00012928, NOTCH2b = EHELV G00012927

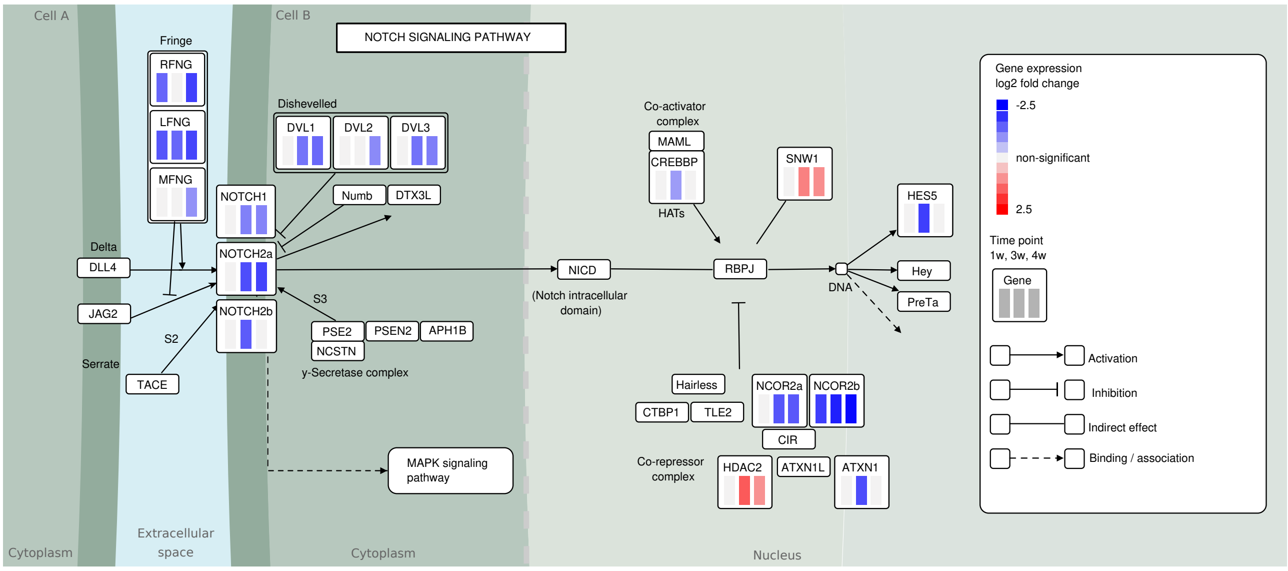

6 Minoru Kanehisa and Susumu Goto. Kegg: kyoto encyclopedia of genes and genomes. Nucleic acids research, 28(1):27–30, 2000.

Figure S13: Heatmaps illustrating overrepresented A) KEGG pathways and B) biological processes when comparing the nipah and Ebola vaccination groups. Ontology terms that were significantly overrepresented (FDR<0.05) are shown in yellow colour, with faint yellow indicating lower fold enrichment score and dark yellow a higher fold enrichment score. GO terms that were not significantly overrepresented are shown in grey for that particular treatment group and time point. Only BPs where at least one group had an enrichment fold of <2 are shown. 1w-4w: blood samples collected weekly

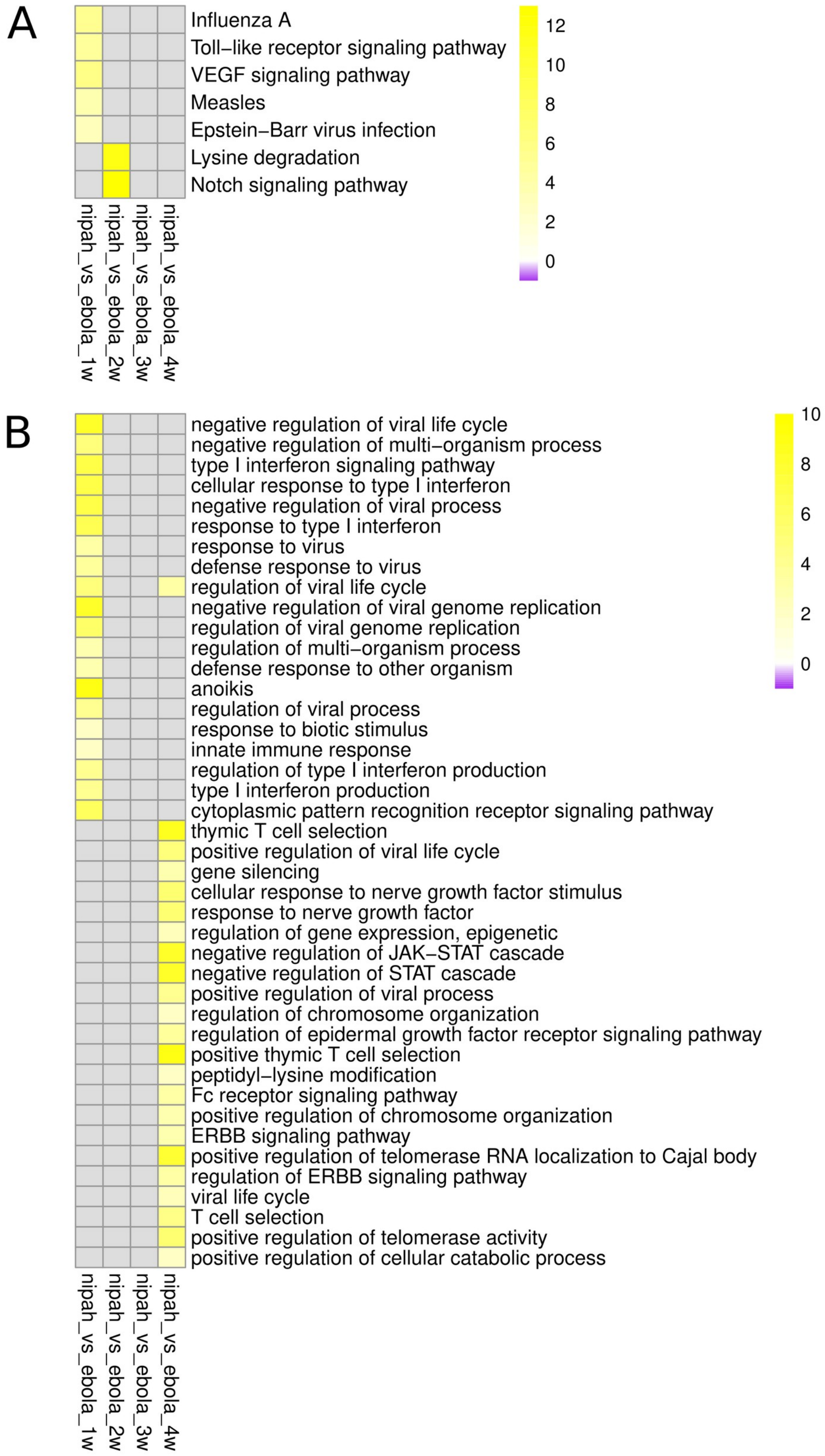

Figure S14: Heatmap illustrating the log2 fold change between the Nipah and Ebola vaccination group of differentially expressed genes from overrepresented antiviral KEGG signaling pathways; A) hsa05164 Influenza A, B) hsa05169 Epstein-Barr virus infection, C) hsa05162 Measles and D) hsa04620 Toll-like receptor signaling pathway. Yellow shading indicates that the gene expression was higher-, and purple shading indicates that the gene expression was lower in bats that received the Ebola vaccine than in bats that received the nipah vaccine. Grey colour indicates time points when the genes were not significantly differentially expressed. Only genes that were differentially expressed in at least one treatment group at one time point are shown. 1w-4w: blood samples collected weekly

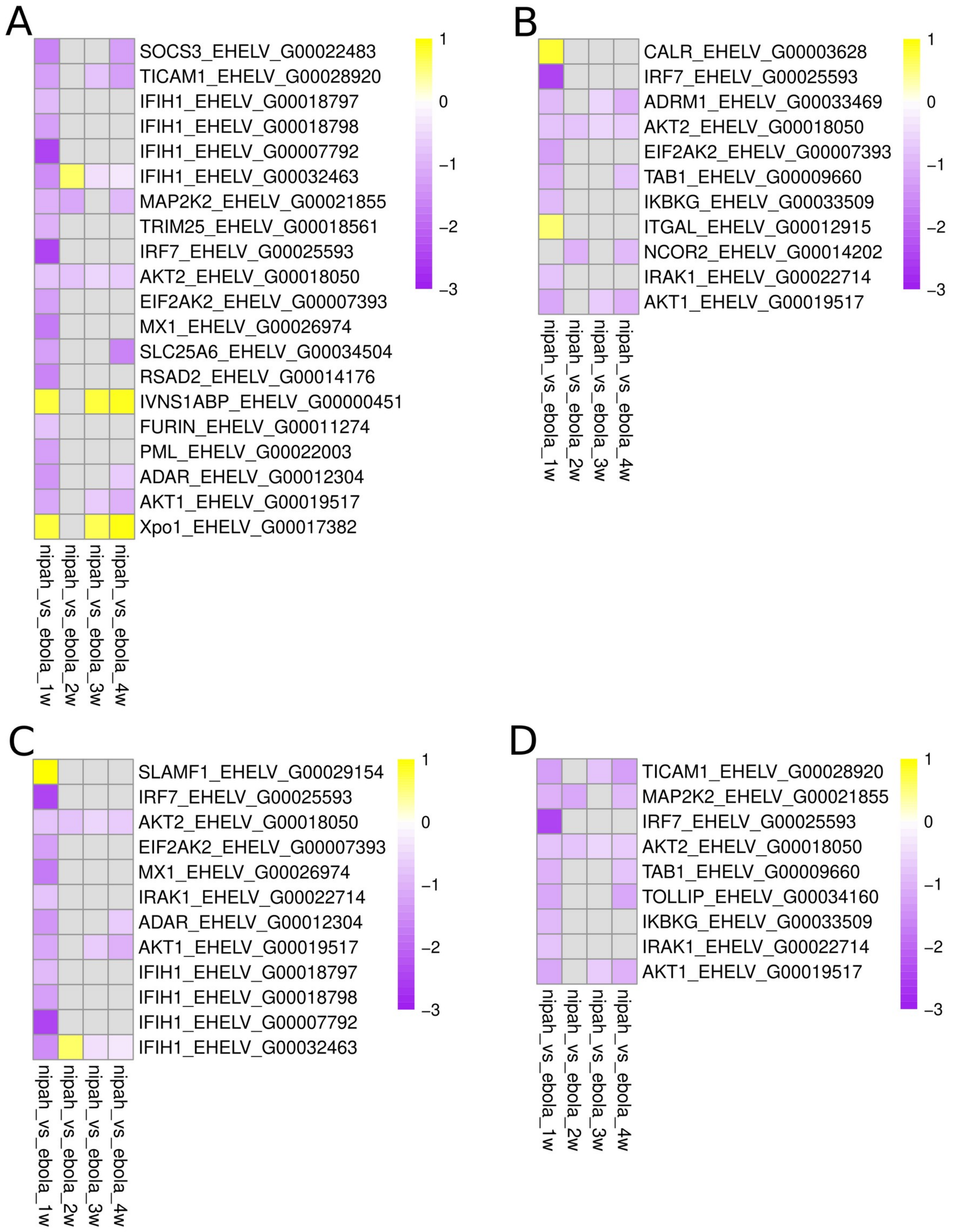

Figure S15: Volcano plot displaying log2 fold change in gene expression between the Nipah and Ebola vaccination groups versus log10 transformed p-value two weeks after vaccination. Genes from overrepresented KEGG pathways (electronic supplementary files, figure S13A) are visualised in colour.

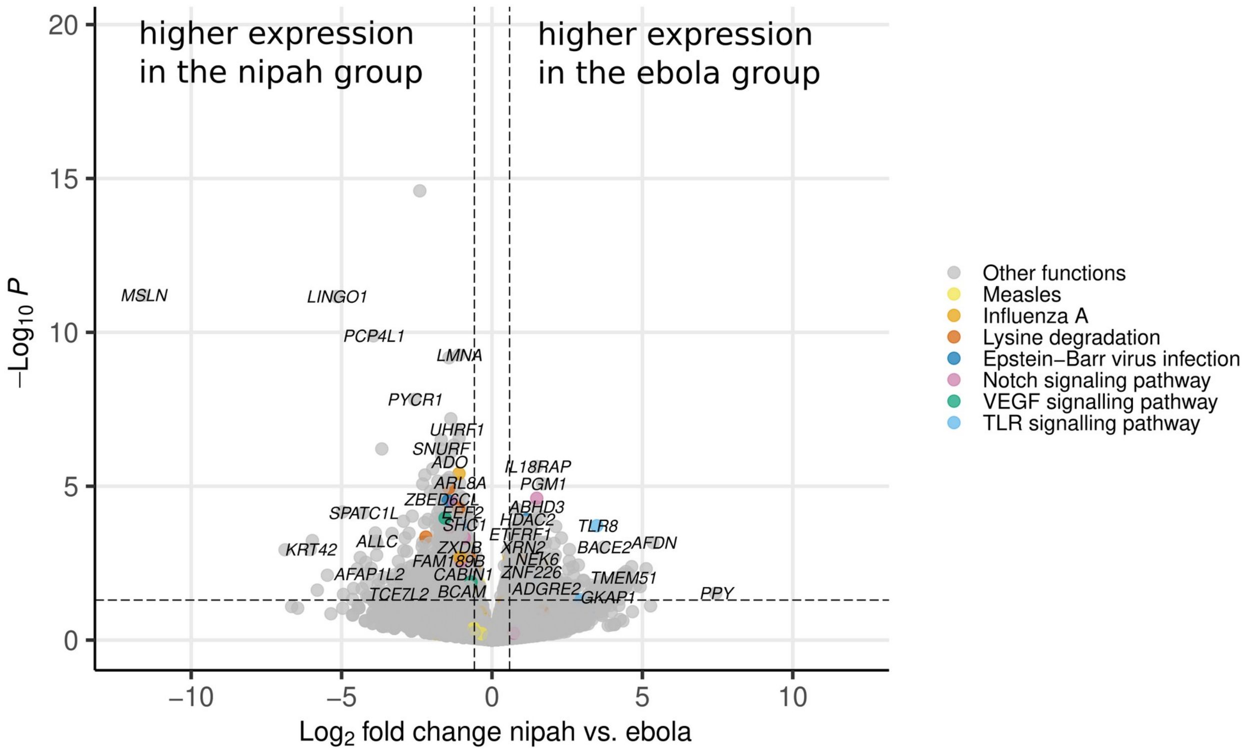

Figure S16: A) Volcano plot displaying log2 fold change in gene expression between the Nipah and Ebola vaccination groups versus log10 transformed p-value three weeks after vaccination. Genes from overrepresented KEGG pathways (electronic supplementary files, figure S13A) are visualised in colour. B) log2 fold change of genes from each treatment group compared to the PBS group. Orange dots denote significantly differentially expressed between the nipah and Ebola vaccination groups, dark orange dots denote significantly differentially expressed genes with a log2 fold change of at least 2.

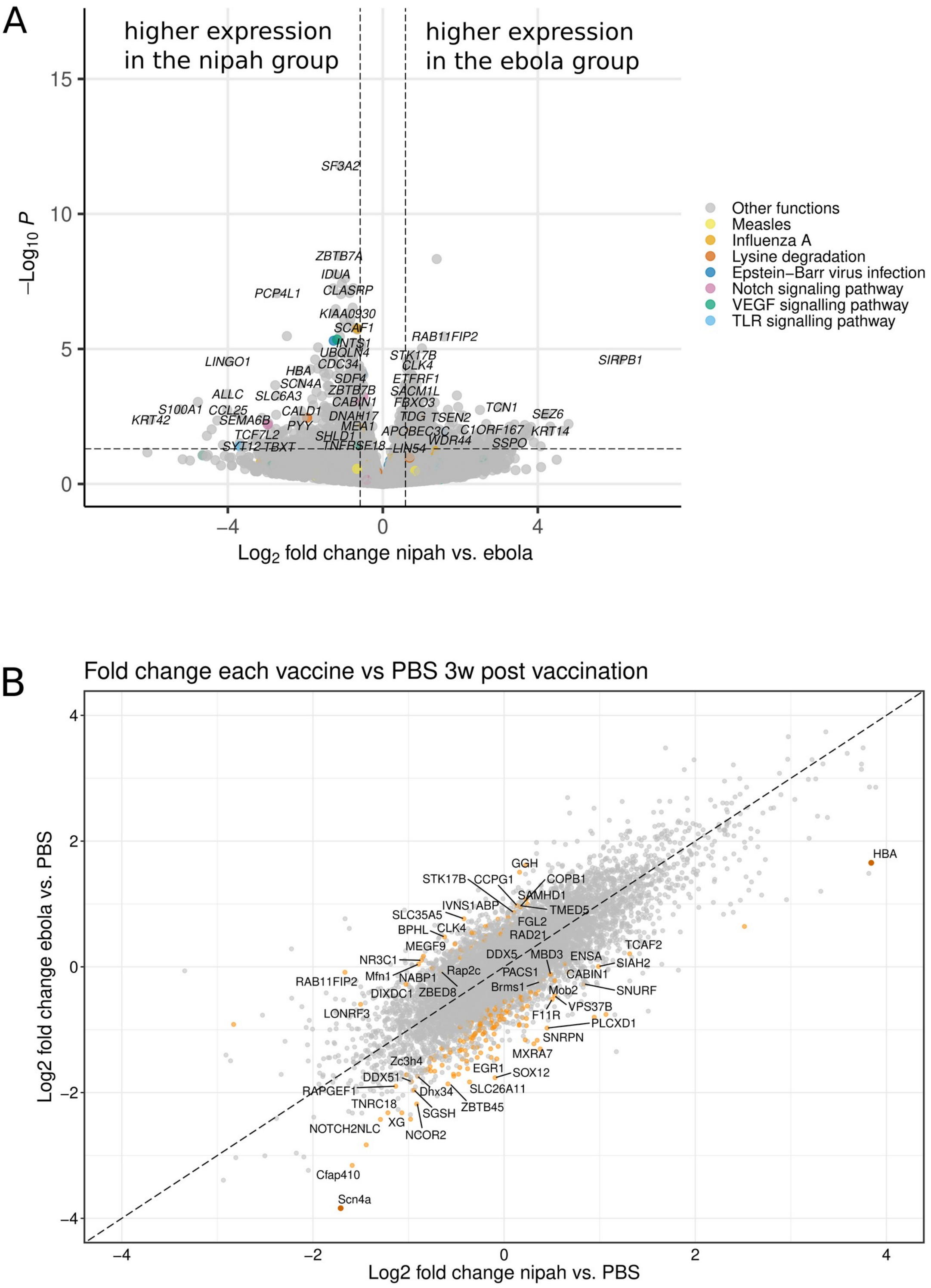

Figure S17: A) Volcano plot displaying log2 fold change in gene expression between the Nipah and Ebola vaccination groups versus log10 transformed p-value four weeks after vaccination. Genes from overrepresented KEGG pathways (electronic supplementary files, figure S13A) are visualised in colour. B) log2 fold change of genes from each treatment group compared to the PBS group. Orange dots denote significantly differentially expressed between the nipah and Ebola vaccination groups, dark orange dots denote significantly differentially expressed genes with a log2 fold change of at least 2, and triangles denote differentially expressed genes up-regulated in one treatment group and down-regulated in the other when compared to the PBS treatment group.

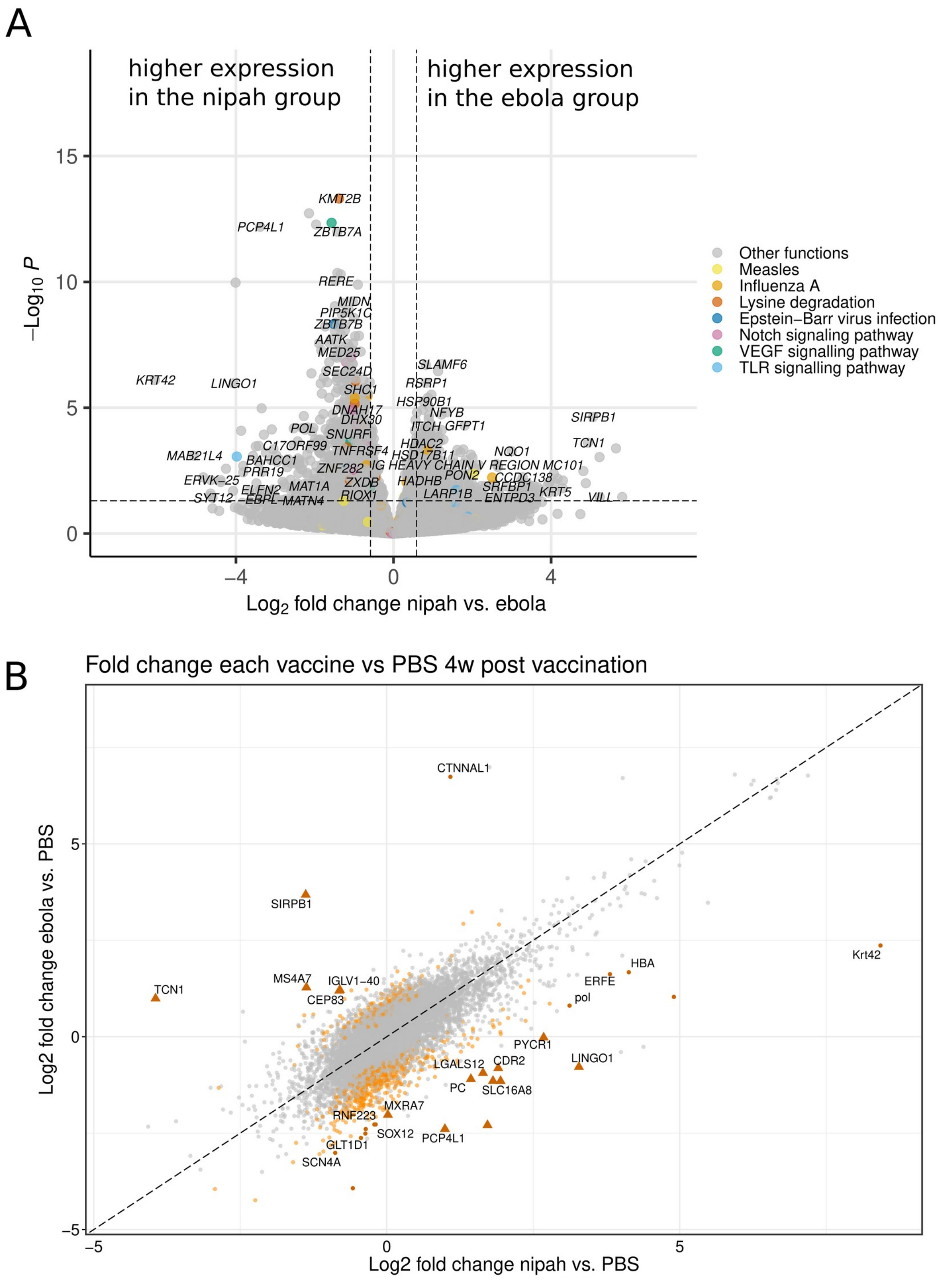

Figure S18: Heatmap illustrating the log2 fold change of the top ten differentially expressed genes (rows) from each timepoint in the comparison between the Nipah and Ebola vaccination groups. Yellow shading indicates that the gene expression was higher-, and purple shading indicates that the gene expression was lower in bats that received the Ebola vaccine than in bats that received the nipah vaccine. Grey colour indicates time points when the genes were not significantly differentially expressed. Genes that could not be assigned a gene name from the *E. helvum* genome are called unknown. All DEGs are listed in electronic supplementary dataset S3.

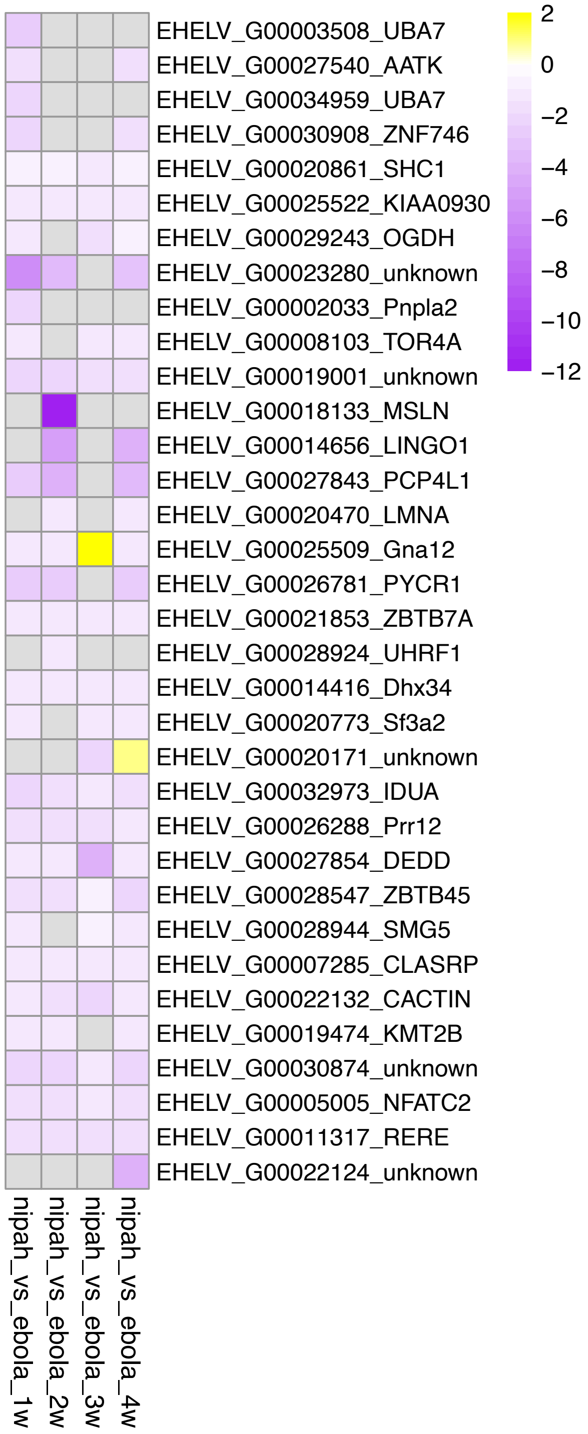

Figure S19. Antigen-Specific MFI Distributions with Plate-Level Negative Controls

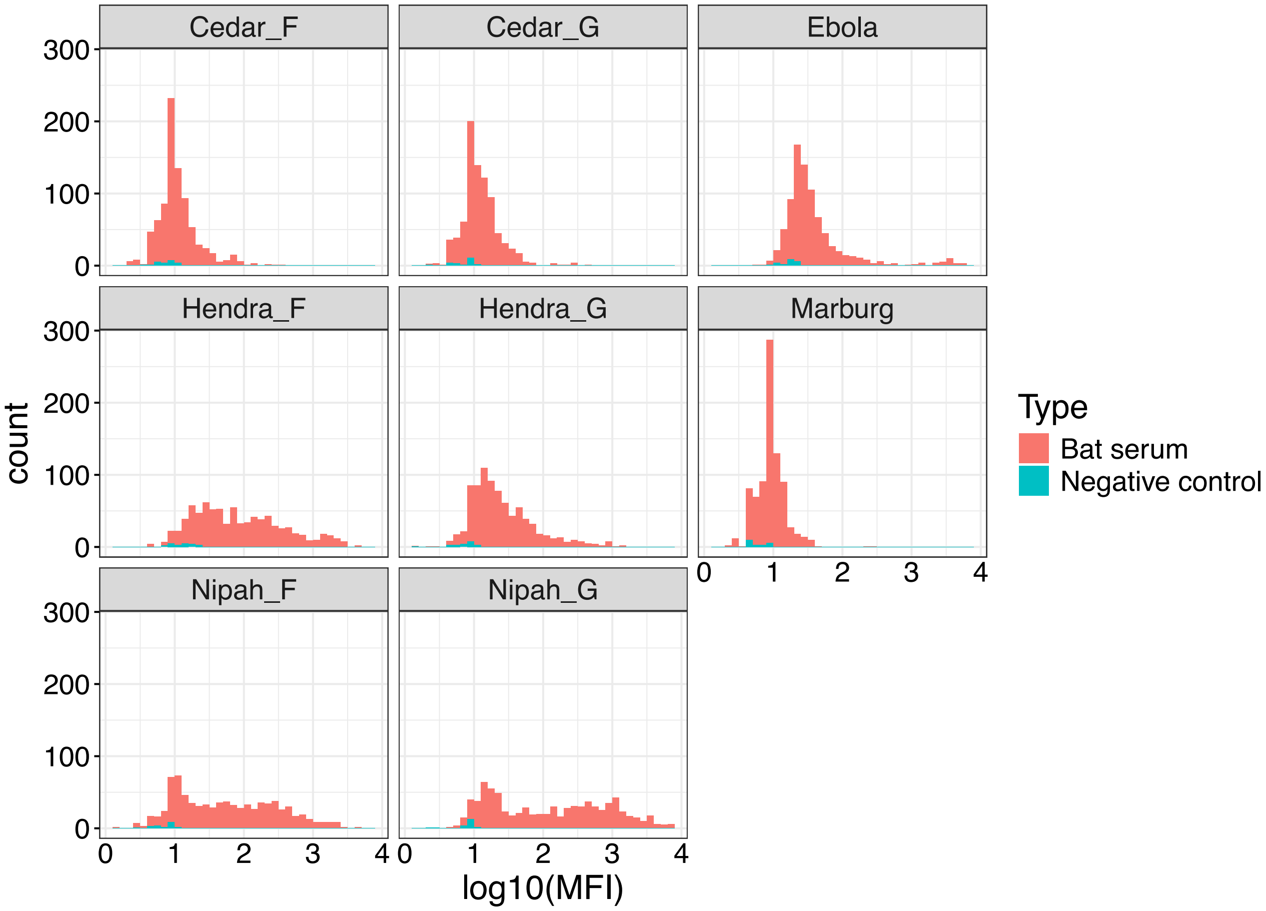

Figure S20: Principal component analysis (PCA) of blood samples collected up to week four (A and B), and following exclusion of individual 5898066 (bottom panels). Each point represent one sample. Shapes indicate the treatment group for each sample, while colour indicate either the sampling time point (left) or the individuals (right) from which the sample was collected. PCA was performed and scatterplots were created using DESeq2. PCA, principal components analysis.

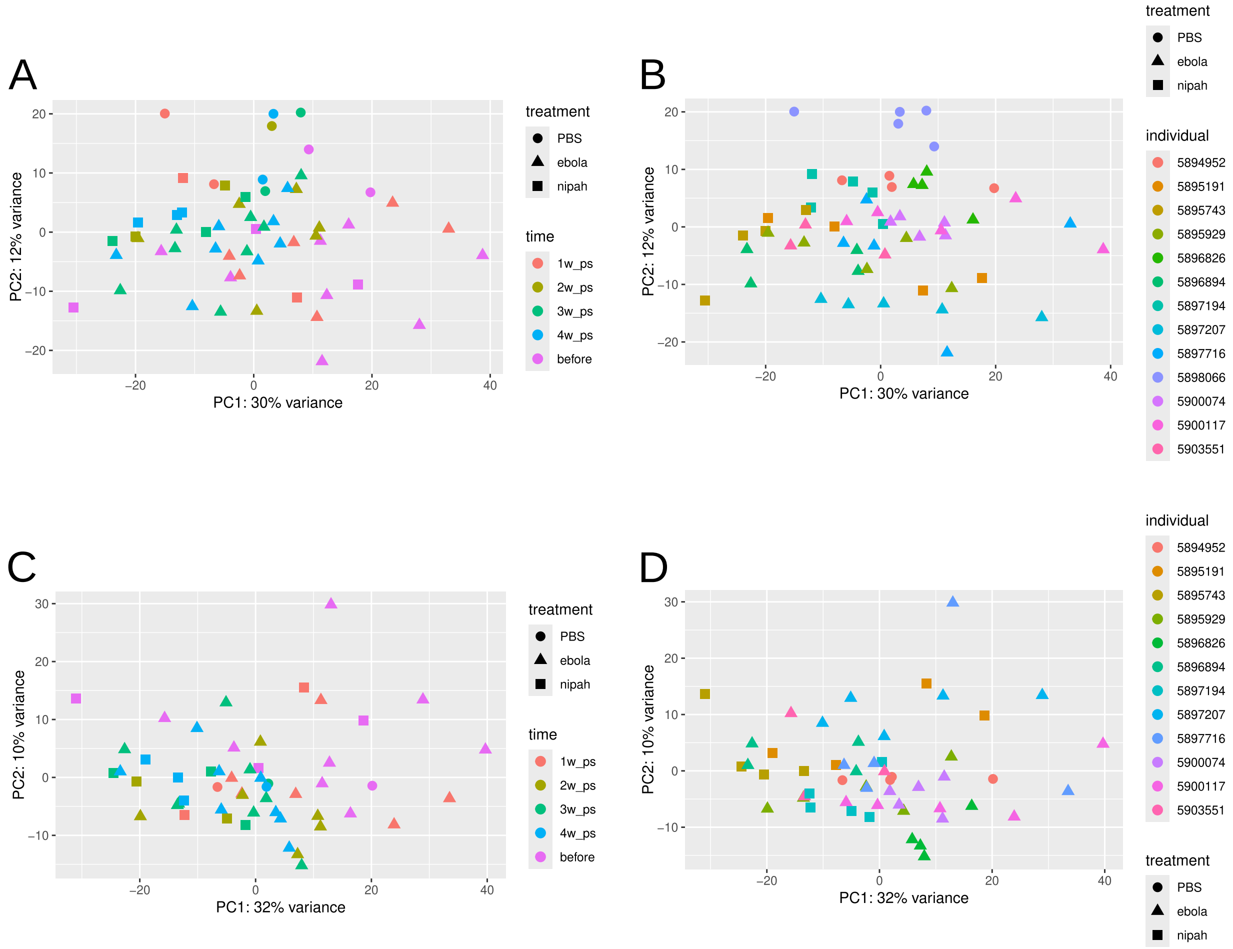

Treatment	before	w1	W2 **	w3	w4
Ebola vaccine	8	6	6	8	8
Nipah vaccine	3	2	2	3	3
PBS *	2 (1)	2 (1)	1 (0)	2 (1)	2 (1)

Table S1. Number of successfully sequenced samples in this study.
* one control individual was excluded from the differential expression analysis due to high levels of Nipah antibodies at the start of the experiment. The number of control individuals included in the differential expression analysis for each time point is showed in brackets.
** no differential expression analysis was done between the vaccination- and the control groups two weeks post vaccination due to lack of control individuals at this time point.

Treatment	Regulation	w1	w2	w3	w4
nipah vs ebola	Upregulated	61	39	45	230
nipah vs ebola	Downregulated	387	161	140	558

Table S2. Number of differentially expressed genes when comparing the nipah and Ebola vaccine groups. The nipah group was used as the reference.

Title: Production of
Stock number: #2352
Produced for: Tess
Quantity Required: standard prep

1.0 SUMMARY

Re-bulk of stock virus **#2277.** The presence of the antigen was confirmed by ID PCR. The virus was titred to obtain infectious units per ml, and assayed by spectrophotometry to measure the number of virus particles per ml. The sterility of the virus was also confirmed by inoculation of TSB broth with 10 µl of purified virus and incubation for 3 days at 35°C. New virus stock **#2352**

Destination vector details: Plasmid no: pC252/p4280
Location of DNAstar Plasmid Map: S:\DNASTAR FILES\Ebola\Shuttle Vectors\ChAdOx1 Shuttle

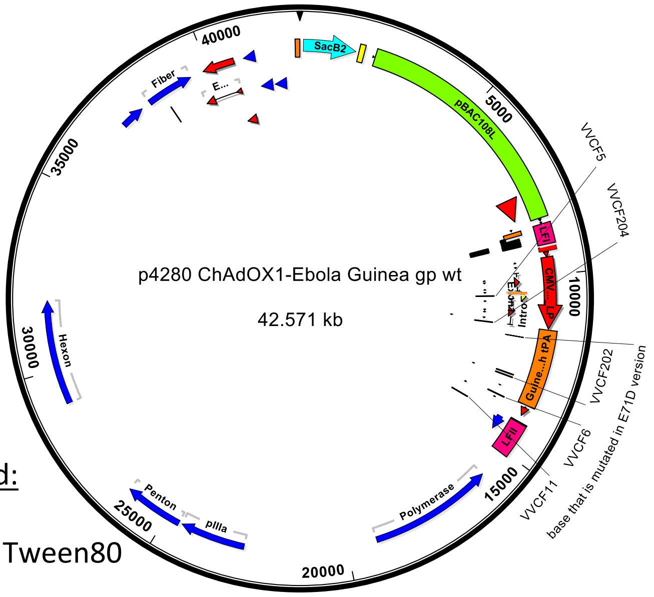

<u>ID PCR</u> **Expected Product size: 1194bp**

VVCF11 GCTCATGATGCTGCAACACAC
VVCF202 GATCACCAACACTATCGCCG

<u>Flank-Flank PCR</u> **Expected Product size: 3151bp**

VVCF5 ATCCACGCTGTTTTGACCTC
VVCF6 CAGCGAGCTCTAGCATTTAGG

<u>NOTE: Made with the following variations to standard method:</u>

Cell pellet resuspended in 10mM Tris pH9.0. 2mM MgCl2. 1% Tween80
Instead of 10mM Tris pH7.8, 1mM MgCl2

Single isopycnic CsCl gradient (2hr) performed. Band extracted and dialysed into:

50mM Tris pH7.8	higher concentration than VVCF normal (10mM)
2mM MgCl2	not present in normal VVCF storage buffer
0.1% PS80	not present in normal VVCF storage buffer
7.5% sucrose	same as normal VVCF storage buffer

ID PCR Conditions

Primary Denature	95°C	3 min	
Denature	95°C	40 sec	X 25
Anneal	60°C	15 sec	
Extension	68°C	2 min	
Final Extension	72°C	3 min	
End			

Flank-Promoter PCR Conditions

Primary Denature	95°C	3 min	
Denature	95°C	40 sec	X 25
Anneal	60°C	15 sec	
Extension	68°C	5 min	
Final Extension	72°C	10 min	
End			

Control virus AdH5-LPTOS-mcherry (stock #1785) Expected Product size: 1807 bp

VVCF5 ATCCACGCTGTTTTGACCTC
VVCF6 CAGCGAGCTCTAGCATTTAGG

Purified virus QC

Identity PCR

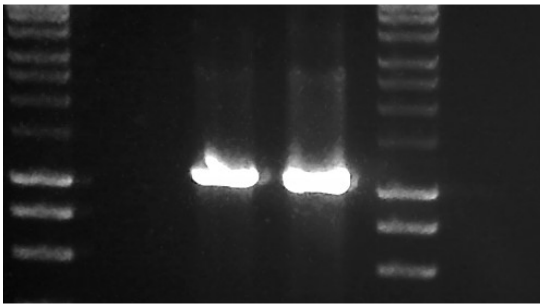

1 2 3 4 5 6

Lane 1	Smartladder (Eurogentech)
Lane 2	sterile water –ve control
Lane 3	pC252 DNA +ve control
Lane 4	#2352 DNA extracted from purified virus
Lane 5	Smartladder (Eurogentech)
Lane 6	Irrelevant virus control

Expected Product size: 1194 bp

Flank PCR

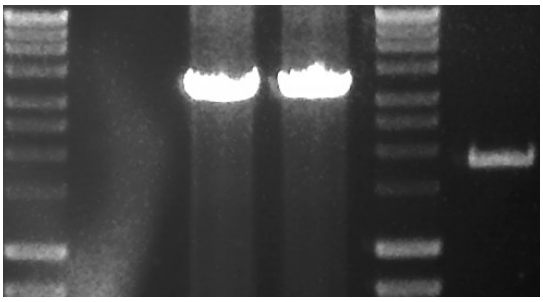

1 2 3 4 5 6

Lane 1	Smartladder (Eurogentech)
Lane 2	sterile water –ve control
Lane 3	pC252 DNA +ve control
Lane 4	#2352 DNA extracted from purified virus
Lane 5	Smartladder (Eurogentech)
Lane 6	Irrelevant virus control

Expected Product size: 3151bp

QC test	Result
Infectious units by titration (IU/ml)	1.7E+10
Virus particle concentration (VP/ml)	4.0E+12
P:I ratio	240
Sterility testing	passed
Location of virus in -80C freezer	At CCMP

Appendix:

Smartladder:

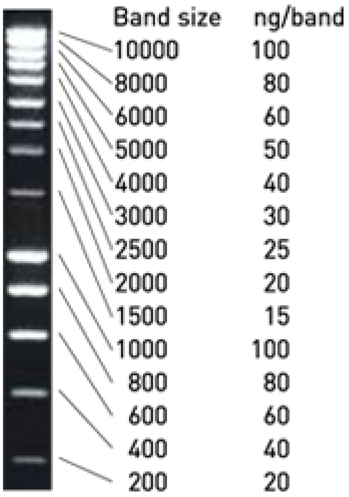

THE JENNER INSTITUTE VIRAL VECTOR CORE FACILITY
THE CENTRE FOR CELLULAR AND MOLECULAR PHYSIOLOGY
ROOSEVELT DRIVE, OXFORD. OX3 7BN

ADENOVIRUS PRODUCTION REPORT

Document Number: VR1067-3

Title: Production of ChAdOX1-Nipah
Stock number: #2842
Produced for: Tess
Quantity Required: std prep

1.0 SUMMARY

Re-bulk of stock virus **#2327.** The presence of the antigen was confirmed by ID PCR. The virus was titred to obtain infectious units per ml, and assayed by spectrophotometry to measure the number of virus particles per ml. The sterility of the virus was also confirmed by inoculation of TSB broth with 10 µl of purified virus and incubation for 3 days at 35°C. New virus stock **#2842**

Material supplied in: 10mM Tris, 7.5% sucrose, 150 mM NaCl, 0.1% Tween80. pH7.8

Destination vector details: Plasmid no: pC557
Location of DNAstar Plasmid Map: S:\DNASTAR FILES\Nipah

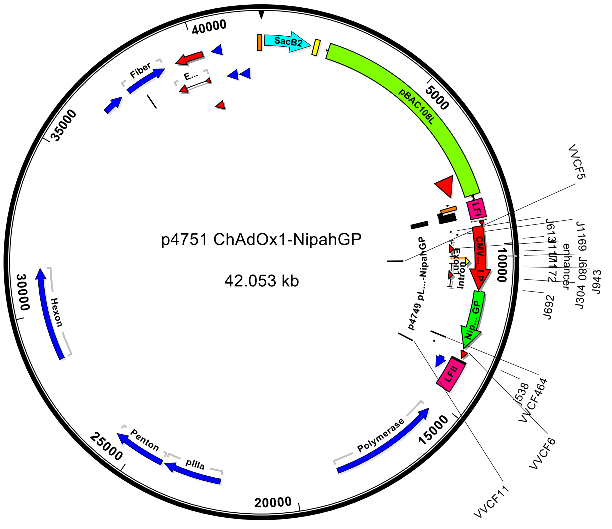

**ID PCR**	**Expected Product size: 635 bp**
VVCF11	GCTCATGATGCTGCAACACAC
VVCF464	gaccatcaccaactgcttcc

**Flank PCR**	**Expected Product size: 2833 bp**
VVCF5	ATCCACGCTGTTTTGACCTC
VVCF6	CAGCGAGCTCTAGCATTTAGG

AdH5-mcherry (#1785) Expected band size 1807 bp

QC test	Result
Infectious units by titration (IU/ml)	1.3E+10
Virus particle concentration (VP/ml)	1.0E+12
P:I ratio	76

ID PCR Conditions

Primary Denature	95°C	3 min	
Denature	95°C	40 sec	X 25
Anneal	60°C	15 sec	
Extension	68°C	1 min	
Final Extension	72°C	3 min	
End			

Flank-Promoter PCR Conditions

Primary Denature	95°C	3 min	
Denature	95°C	40 sec	X 25
Anneal	60°C	15 sec	
Extension	68°C	3 min	
Final Extension	72°C	5 min	
End			

Control virus AdH5-LPTOS-mcherry (stock #1785) Expected Product size: 1807 bp
VVCF5 ATCCACGCTGTTTTGACCTC
VVCF6 CAGCGAGCTCTAGCATTTAGG

Purified virus QC

Identity PCR

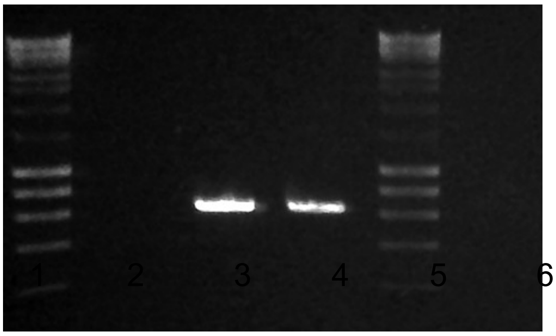

Lane 1 Smartladder (Eurogentech)
Lane 2 sterile water –ve control
Lane 3 plasmid DNA +ve control
Lane 4 DNA extracted from purified virus
Lane 5 Smartladder (Eurogentech)
Lane 6 Irrelevant virus control

Expected Product size: 635 bp

Flank PCR

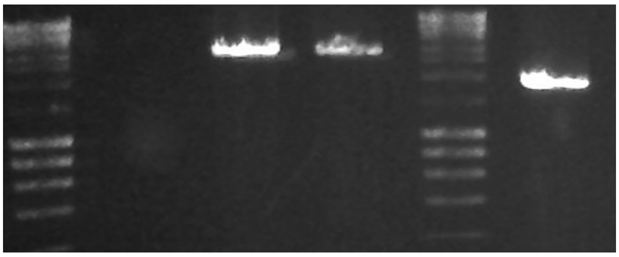

1 2 3 4 5 6

Lane 1 Smartladder (Eurogentech)
Lane 2 sterile water –ve control
Lane 3 plasmid DNA +ve control
Lane 4 DNA extracted from purified virus
Lane 5 Smartladder (Eurogentech)
Lane 6 Irrelevant virus control

Expected Product size: 2833 bp

THE JENNER INSTITUTE VIRAL VECTOR CORE FACILITY
OLD ROAD CAMPUS RESEARCH BUILDING, ROOSEVELT DRIVE,
OXFORD OX3 7DQ

MVA PRODUCTION REPORT

Document Number:VR1067-2

Title: Production of MVA-WT-Zaire
Stock number: #2841
Produced for: Tess
Prep size required:standard prep

1.0 SUMMARY

Re-bulk of stock **premaster #1858.** To confirm the presence of the antigen in question and lack of red virus contamination in the final stock, ID and purity PCR were performed. The virus was also titred. The sterility of the virus was also confirmed by inoculation of TSB broth with 10 µl of purified virus and incubation for 3 days at 35°C. New virus stock **#2841.**

Recombinant MVA vectors are supplied in **10 mM Tris, 140 mM NaCl, pH 7.8.**

Shuttle vector details: Plasmid no. p4284/pC224

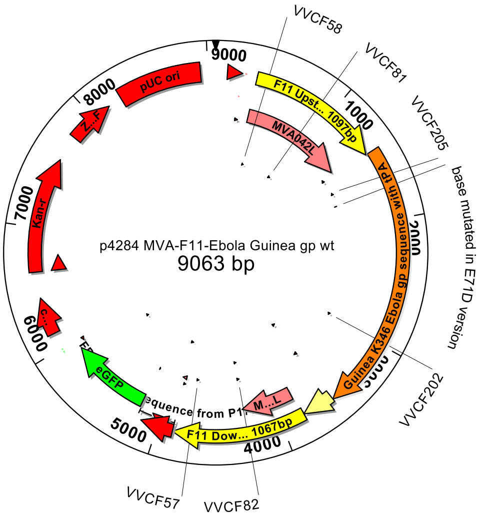

ID PCR **Expected band size 1323 bp**

**Name**	**Sequence**
VVCF202	GATCACCAACACTATCGCCG
VVCF82	ATCCCATGGCCCACGCACACAC

Flank PCR **Expected band size 3419 bp**

VVCF81	CAATACCCTGCAGGACGTCCCAACTAC
VVCF82	ATCCCATGGCCCACGCACACAC

(VVCF81-VVCF82 MVA.F-11 dsRED expected band 1976 bp)

Purity:

VVCF82	ATCCCATGGCCCACGCACACAC
VVCF4	GCTGAAGGGCGAGATCCACAAGGC

(Product size for MVA F11 dsRED: 945 bp)

Control Virus: Stock # 1597

QC test	Result
Plaque forming units by titration (PFU/ml)	1.2E+9
Sterility testing	passed

ID PCR Conditions

PCR to confirm the presence of the antigen. The antigen-specific primer binds towards the 3′ end of the gene and therefore does not confirm the integrity of the antigen upstream of this site.

Primary Denature	95°C	3 min	
Denature	95°C	40sec	X 20
Anneal	60°C	15 sec	
Extension	68°C	2 min	
Final Extension	72°C	5 min	
End			

Flank-Promoter PCR Conditions

PCR covering the length of the antigen insertion site. This will confirm the integrity of the antigen as far as the resolution of the agarose gel will permit. This PCR will also identify any cross contamination with vectors containing the p7.5 promoter as far as detectable by this method.

Primary Denature	95°C	3 min	
Denature	95°C	40sec	X 20
Anneal	60°C	15 sec	
Extension	68°C	4 min	
Final Extension	72°C	7 min	
End			

Purity summary:

PCR to confirm the absence of parental MVA-RFP to the limit of detection of this PCR. This PCR will also detect the transient stage of MVA during marker-less virus production

Primary Denature	95°C	3 min	
Denature	95°C	40sec	X 20
Anneal	60°C	15 sec	
Extension	68°C	2 min	
Final Extension	72°C	3 min	
End			

THE JENNER INSTITUTE VIRAL VECTOR CORE FACILITY
THE CENTRE FOR CELLULAR AND MOLECULAR PHYSIOLOGY
ROOSEVELT DRIVE, OXFORD. OX3 7BN

MVA PRODUCTION REPORT

Document Number: VR1068-4

Title: Production of
Stock number:
Produced for:
Prep size required:

1.0 SUMMARY

Re-bulk of stock **premaster #2265.** To confirm the presence of the antigen in question and lack of red virus contamination in the final stock, ID and purity PCR were performed. The virus was also titred. The sterility of the virus was also confirmed by inoculation of TSB broth with 10 µl of purified virus and incubation for 3 days at 35°C.

Virus supplied in formulation buffer: **10 mM Tris, 140 mM NaCl, pH 7.8.**

Shuttle vector details: Plasmid no. p434/pC578
Location of DNAstar file: S:\DNASTAR FILES\vectors\poxvirus vectors\MVA-GFP

ID PCR not performed – no antigen present in normal antigen insertion site. GFP expression was confirmed visually during production (PCR was not performed to look for it).

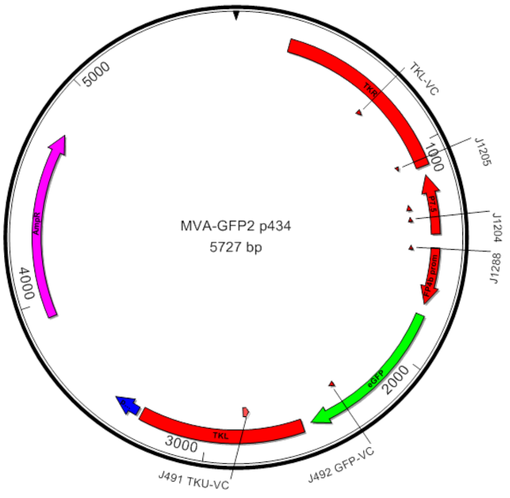

Flank-Flank PCR **Expected band size 1770 bp**

VVCF91 AACGGCGGACATATTCAGTT
VVCF2 CCATCGAGTGCGGCTACTAT
(VVCF1-2 MVA.RED expected band 977 bp)

Purity: **Product size for MVA_RFP: 637 bp**
VVCF55 GCGACCTCATTTGCACTTTCTGGTTCG
VVCF4 GCTGAAGGGCGAGATCCACAAGGC

Parental virus: #2269

QC test	Result
Plaque forming units by titration (PFU/ml)	9.7E+8
Sterility testing	passed

ID PCR Conditions

PCR to confirm the presence of the antigen. The antigen-specific primer binds towards the 3´ end of the gene and therefore does not confirm the integrity of the antigen upstream of this site.

Primary Denature	95°C	3 min	
Denature	95°C	40 sec	X 20
Anneal	60°C	15 sec	
Extension	68°C	1 min	
Final Extension	72°C	3 min	
End			

Flank-Promoter PCR Conditions

PCR covering the length of the antigen insertion site. This will confirm the integrity of the antigen as far as the resolution of the agarose gel will permit. This PCR will also identify any cross contamination with vectors containing the p7.5 promoter as far as detectable by this method.

Primary Denature	95°C	3 min	
Denature	95°C	40sec	X 20
Anneal	60°C	15 sec	
Extension	68°C	3 min	
Final Extension	72°C	5 min	
End			

Purity summary:

PCR to confirm the absence of parental MVA-RFP to the limit of detection of this PCR. This PCR will also detect the transient stage of MVA during marker-less virus production

Primary Denature	95°C	3 min	
Denature	95°C	40sec	X 20
Anneal	60°C	15 sec	
Extension	68°C	1 min	
Final Extension	72°C	3 min	
End			
